# Development of the First ^18^F-Labeled Radiohybrid-Based Minigastrin Derivative with High Target Affinity and Tumor Accumulation by Substitution of the Chelating Moiety

**DOI:** 10.3390/pharmaceutics15030826

**Published:** 2023-03-03

**Authors:** Thomas Günther, Nadine Holzleitner, Daniel Di Carlo, Nicole Urtz-Urban, Constantin Lapa, Hans-Jürgen Wester

**Affiliations:** 1 Pharmaceutical Radiochemistry, Technical University of Munich, 85748 Garching, Germany; 2 Nuclear Medicine, Faculty of Medicine, University of Augsburg, 86156 Augsburg, Germany

**Keywords:** cholecystokinin-2 receptor (CCK-2R), cholecystokinin-B receptor (CCK-BR), MTC, ^18^F-labeled minigastrin analogs, radiohybrid, rhCCK

## Abstract

In order to optimize elevated kidney retention of previously reported minigastrin derivatives, we substituted (*R*)-DOTAGA by DOTA in (*R*)-DOTAGA-rhCCK-16/-18. CCK-2R-mediated internalization and affinity of the new compounds were determined using AR42J cells. Biodistribution and *µ*SPECT/CT imaging studies at 1 and 24 h p.i. were carried out in AR42J tumor-bearing CB17-SCID mice. Both DOTA-containing minigastrin analogs exhibited 3- to 5-fold better *IC*_50_ values than their (*R*)-DOTAGA-counterparts. ^nat^Lu-labeled peptides revealed higher CCK-2R affinity than their ^nat^Ga-labeled analogs. In vivo, tumor uptake at 24 h p.i. of the most affine compound, [^19^F]F-[^177^Lu]Lu-DOTA-rhCCK-18, was 1.5- and 13-fold higher compared to its (*R*)-DOTAGA derivative and the reference compound, [^177^Lu]Lu-DOTA-PP-F11N, respectively. However, activity levels in the kidneys were elevated as well. At 1 h p.i., tumor and kidney accumulation of [^19^F]F-[^177^Lu]Lu-DOTA-rhCCK-18 and [^18^F]F-[^nat^Lu]Lu-DOTA-rhCCK-18 was high. We could demonstrate that the choice of chelators and radiometals has a significant impact on CCK-2R affinity and thus tumor uptake of minigastrin analogs. While elevated kidney retention of [^19^F]F-[^177^Lu]Lu-DOTA-rhCCK-18 has to be further addressed with regard to radioligand therapy, its radiohybrid analog, [^18^F]F-[^nat^Lu]Lu-DOTA-rhCCK-18, might be ideal for positron emission tomography (PET) imaging due to its high tumor accumulation at 1 h p.i. and the attractive physical properties of fluorine-18.

## 1. Introduction

In 2022, an estimated number of 43,800 new thyroid cancer cases will occur in the United States [1]. Out of these, medullary thyroid carcinoma (MTC) comprises only 2–3% and thus rarely occurs. However, due to comparably late tumor detection in advanced stages and limited treatment options of this disease, the 5- and 10-year survival of MTC (65–89% and 71–87%, respectively) is lower compared to that of the more common types of differentiated thyroid cancer [2,3]. The 10-year survival rate for patients developing metastatic MTC is only 10%, which underlines the importance of an early diagnosis as well as novel therapeutic options [4].

In contrast to conventional diagnostic methodologies, nuclear medicine provides an opportunity to exhibit biochemical information through non-invasive molecular imaging applications. Amongst other uses, this allows for the localization of tumor lesions and metastases because most malignant cells overexpress certain target structures that can be addressed by radiopharmaceutical drugs. In the case of MTC, over 90% of tumors overexpress the cholecystokinin-2 receptor (CCK-2R) in high density [5]. In spite of this characteristic, the gold standard for MTC imaging is ^18^F-based positron emission tomography (PET) using [^18^F]F-DOPA (3,4-dihydroxy-6-[^18^F]fluoro-L-phenylalanine) instead of a CCK-2R-targeted compound [6]. This can be attributed to the lack of an efficient ^18^F-labeled CCK-2R-targeted compound, which could combine the favorable properties of fluorine-18 and PET alongside targeting the CCK-2R, as well as the fact that [^18^F]F-DOPA is already clinically established for neuroimaging [7,8,9]. Because [^18^F]F-DOPA is trapped in neuroendocrine tumor cells such as MTC due to the availability of an excess of aromatic L-amino acid decarboxylase (AADC) in these cells [7,10,11], a sensitivity of 86% was observed for primary MTC [12]. However, only moderate sensitivity was determined for [^18^F]F-DOPA-PET in both lymph node metastases and distant metastases [10,13].

In 2021, Khan et al. reported the first attempts to introduce scaffolds into the peptide structure of the CCK-2R-targeting minigastrin analog MG11 (glu-Ala-Tyr-Gly-Trp-Met-Asp-Phe-NH_2_) that enables direct radiofluorination via a nucleophilic aromatic substitution of a nitro group by [^18^F]fluoride (K_2_CO_3_, kryptofix 2.2.2, azeotropic drying). However, rapid de-fluorination due to the poor chemical stability of these compounds was observed, which illustrates the need for optimized alternatives [14]. In addition, the earlier studies of Good et al. demonstrated a poor metabolic stability for MG11 in vivo [15,16], aggravating the use of this basic structure as a scaffold for radiopharmaceuticals, which is why alternative strategies are desired.

In a previous study, we addressed this topic by introducing a silicon-based fluoride acceptor (SiFA) moiety into the peptide structure of DOTA-PP-F11N, a minigastrin analog that is currently in clinical trials. However, one of the biggest limitations of [^177^Lu]Lu-DOTA-PP-F11N represents its high hydrophilicity and thus fast clearance kinetics [17]. In contrast, the introduction of the SiFA building block enables facile ^18^F-labeling via an isotopic exchange reaction, which is chemically stable [18]. Conversly, the highly lipophilic SiFA moiety compensates for the high hydrophilic character of the peptide. The presence of a SiFA and a chelator moiety within the same molecule allows for either ^18^F- or ^177^Lu-labeling generating chemically identical agents, which are called radiohybrids (rh). Previous rhCCK derivatives, [^19^F]F-[^177^Lu-]Lu-(*R*)-DOTAGA-rhCCK-9 and -16, revealed 3- to 8-fold increased activity levels in the tumor compared to [^177^Lu]Lu-DOTA-PP-F11N at 24 h p.i., respectively, despite a noticeably lower CCK-2R affinity. Nevertheless, activity uptake and retention in the kidneys was substantially elevated, which was likely due to the numerous negative charges in proximity to the SiFA moiety [19].

**Figure 1:**
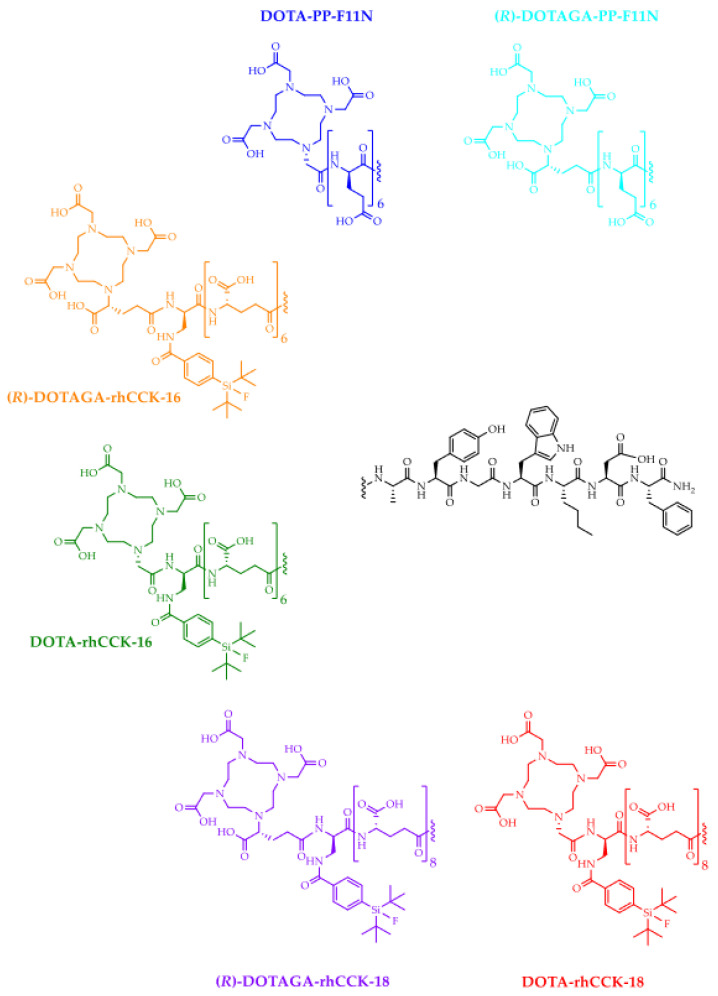
Chemical structures of the (*R*)-DOTAGA- and DOTA-comprising minigastrin analogs evaluated in this study.

Hence, in this study, we wanted to maintain high activity levels in the tumor while reducing elevated kidney retention. Because we aimed to retain the peptide structure (*H*-(γ-glu)_6–8_-Ala-Tyr-Gly-Trp-Nle-Asp-Phe-NH_2_), we only substituted (*R*)-DOTAGA (2-(4,7,10-tris(carboxymethyl)-1,4,7,10-tetraazacyclododecan-1-6yl)pentanedioic acid) by the DOTA (1,4,7,10-tetraazacyclododecane-1,4,7,10- tetraacetic acid) chelator to reduce the negative charge distribution in the direct neighborhood to the SiFA moiety. The resulting analogs (Figure 1) were evaluated by state-of-the-art experiments (*IC*_50_, log*D*_7.4_, receptor-mediated internalization, biodistribution and imaging studies).

## 2. Materials and Methods

Characterization of all CCK-2R-targeted compounds is provided in the Supplementary Materials (Figures S1–S17). Electrospray ionization mass spectrometry for characterization of the substances were acquired on an expression^L^ CMS mass spectrometer (Advion Ltd., Harlow, UK).

### 2.1. Chemical Synthesis and Labeling Procedures

Synthesis of all compounds was conducted via standard Fmoc-based solid phase peptide synthesis (SPPS) on a *H-*Rink Amide ChemMatrix^®^ resin (35–100 mesh particle size, 0.4–0.6 mmol/g loading, Merck KGaA, Darmstadt, Germany) either manually or with a Liberty Blue peptide synthesizer (*H-*Rink Amide ProTide resin, 100–200 mesh particle size, 0.6–0.8 mmol/g loading, CEM GmbH, Stuttgart, Germany). Purification of the peptide precursors was carried out by reversed-phase high-performance liquid chromatography (RP-HPLC). Labeling with [^nat/177^Lu]lutetium or [^nat^Ga]gallium was performed as previously published [20,21]. A detailed description of ^18^F-labeling is provided in the Supplementary Materials. Briefly, ^18^F-fluorination of [^nat^Lu]Lu-DOTA-rhCCK-18 (1 nmol) was conducted via an isotopic exchange reaction at the SiFA building block at 60°C for 5 min using previously dried [^18^F]fluoride (approx. 400 MBq). Afterwards, the ^18^F-labeled peptide was purified via an Oasis^®^ HLB (30 mg) Light Cartridge (Waters GmbH, Eschborn, Germany).

### 2.2. In Vitro Experiments

Cell-based experiments and determination of lipophilicity (log*D*_7.4_) were performed as previously published [19]. A detailed description of the in vitro experiments is provided in the Supplementary Materials.

Human serum albumin (HSA) binding was determined in analogy to a previously published ultracentrifugation method [22]. Therefore, the peptides of interest were incubated in a solution of HSA (700 µM in phosphate-buffered saline) at 37 °C for 30 min (*n* = 6). All values were corrected for unspecific binding.

In vitro stability studies in human serum after incubation at 37 °C for 24 h were performed as described in the Supplemental Material.

### 2.3. In Vivo Experiments

All animal experiments were conducted in accordance with general animal welfare regulations in Germany (German animal protection act, in the edition of the announcement, dated 18 May 2006, as amended by Article 280 of 19 June 2020, approval no. ROB-55.2-1-2532.Vet_02-18-109 by the General Administration of Upper Bavaria) and the institutional guidelines for the care and use of animals. CB17-SCID mice of both genders and aged 2–12 months (Charles River Laboratories International Inc., Sulzfeld, Germany) were allowed to acclimate at the in-house animal facility for at least one week before inoculation was performed. Tumor xenografts were established as previously reported [19]. Exclusion criteria for animals from an experiment were either weight loss higher than 20%, a tumor size above 1500 mm^3^, an ulceration of the tumor, respiratory distress or a change of behavior. None of these criteria applied to any animal from the experiment. Neither randomization nor blinding was applied in the allocation of the experiments. Health status is SPF according to FELASA recommendation.

Biodistribution studies (*n* = 4) and *µ*SPECT/CT as well as *µ*PET/CT imaging (using a MILabs VECTor^4^ small-animal SPECT/PET/OI/CT device, MILabs, Utrecht, The Netherlands) at 1 and 24 h p.i. were carried out as previously published [19,23]. For all ^177^Lu-labeled compounds, approximately 2–3 MBq (100 pmol)—and for the ^18^F-labeled analog, approximately 7 MBq (100 pmol)—were administered. For all competition studies, 2.90 mg/kg (40 nmol) of [^nat^Lu]Lu-DOTA-MGS5 (10^−3^ M in phosphate-buffered saline) were co-administered.

Acquired data were statistically analyzed by performing a Student’s *t*-test via Excel (Microsoft Corporation, Redmond, WA, USA) and OriginPro software (version 9.7) from OriginLab Corporation (Northampton, MA, USA). Acquired *p* values of <0.05 were considered statistically significant.

## 3. Results

### 3.1. Synthesis and Radiolabeling

The precursors were synthesized via standard Fmoc-based SPPS with subsequent RP-HPLC purification in yields of 5–20% (chemical purity >95%, determined by RP-HPLC at λ = 220 nm). Labeling with [^nat^Lu]lutetium as well as [^nat^Ga]gallium was achieved in quantitative yields using a 2.5-fold excess of LuCl_3_ and Ga(NO_3_)_3_, respectively. No purification step was performed because an excess of free ions was not shown to have any impact on overall affinity data [23]. All compounds were labeled manually with lutetium-177, resulting in quantitative radiochemical yields and purities (RCYs, RCPs) and molar activities (A_m_) of 10–50 GBq/µmol. After radiolabeling, no further purification steps were conducted. ^18^F-Labeling of [^nat^Lu]Lu-DOTA-rhCCK-18 was performed manually at 60°C for 5 min. After purification of the ^18^F-labeled peptide via an Oasis^®^ HLB (30 mg) Light Cartridge, RCYs (without further optimization) of 10–30% and molar activities of A_m_ ~85 GBq/µmol and RCPs > 95% were achieved.

### 3.2. In Vitro Characterization

**Figure 2:**
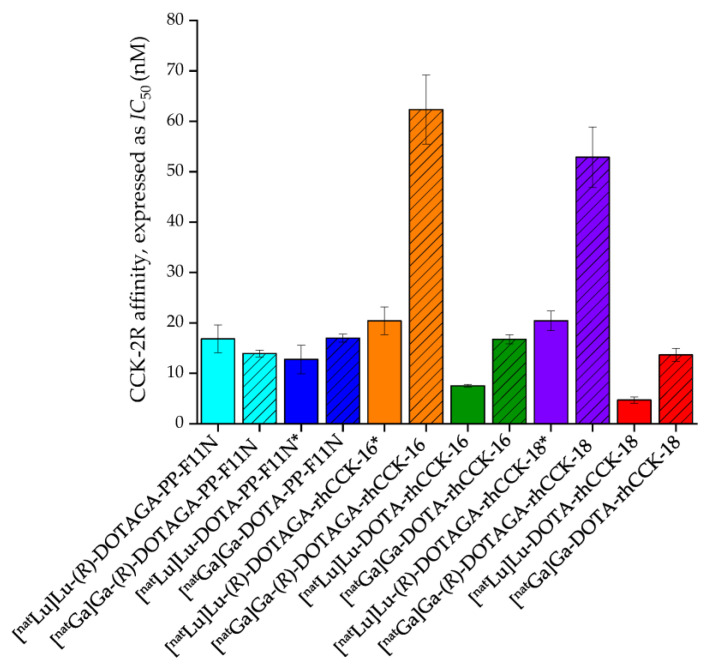
CCK-2R affinity (expressed as *IC*_50_) of the reference compound DOTA-PP-F11N in comparison to rhCCK-16 and -18 containing either a DOTA or (*R*)*-*DOTAGA moiety labeled either with [^nat^Ga]gallium (hatched bars) or [^nat^Lu]lutetium. *IC*_50_ values were determined using AR42J cells (2.0 × 10^5^ cells per well) and [^177^Lu]Lu-DOTA-PP-F11N (0.3 pmol/well) as the radiolabeled reference (3 h, 37 °C, RPMI 1640, 5 mm L-Gln, 5 mL non-essential amino acids (100×), 10% fetal calf serum (FCS) + 5% bovine serum albumin (BSA) (*v*/*v*)). * data taken from Holzleitner et al. [19]. These data have been determined in our lab under identical conditions.

The affinity data of all compounds evaluated are outlined in Figure 2 and Table S1.

In comparison to their (*R*)-DOTAGA-comprising counterparts, all DOTA-containing ligands revealed lower *IC*_50_ values, except for [^nat^Ga]Ga-(*R*)-DOTAGA-PP-F11N. Furthermore, the ^nat^Lu-labeled compounds exhibited higher CCK-2R affinity than their ^nat^Ga-labeled derivatives, except for [^nat^Ga]Ga-(*R*)-DOTAGA-PP-F11N. Overall, [^nat^Lu]Lu-DOTA-rhCCK-18 exhibited the highest CCK-2R affinity among all compounds, and its *IC*_50_ value was 2-fold lower compared to the reference, [^nat^Lu]Lu-DOTA-PP-F11N.

Lipophilicity (log*D*_7.4_) and human serum albumin (HSA) binding data are summarized in Table 1.

In general, all compounds containing a (*R*)-DOTAGA chelator revealed a significantly higher lipophilicity than their DOTA-comprising counterparts (*p* < 0.002). Furthermore, all rhCCK derivatives that comprise a SiFA moiety displayed a distinctly higher lipophilicity (log*D*_7.4_ > −2.7) compared to the reference, [^177^Lu]Lu-DOTA-PP-F11N, and its (*R*)-DOTAGA-containing analog (log*D*_7.4_ < −4.0, *p* < 0.001). Similar log*D*_7.4_ values were found for the chemically identical compounds [^19^F]F-[^177^Lu]Lu-DOTA-rhCCK-18 and [^18^F]F-[^nat^Lu]Lu-DOTA-rhCCK-18 (*p* > 0.22). In addition, HSA binding was observed to be increased for [^177^Lu]Lu-DOTA-rhCCK-16 (89.1%) compared to [^177^Lu]Lu-DOTA-rhCCK-18 (62.6%).

**Figure 3:**
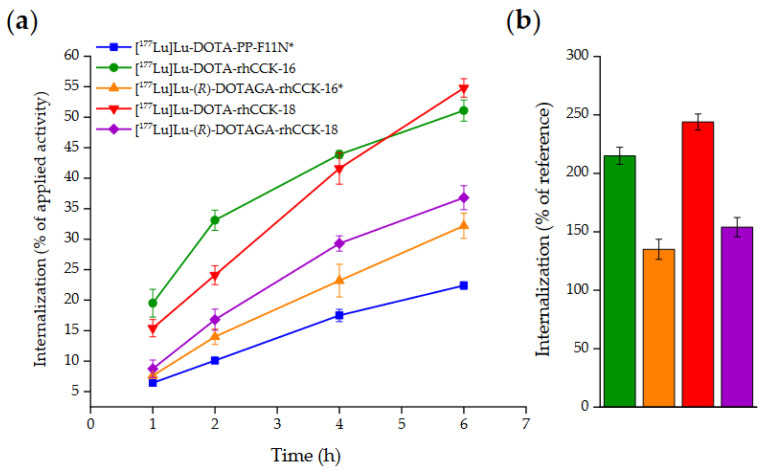
(**a**) CCK-2R-mediated internalization (0.25 pmol/well) measured on AR42J cells as percent (%) of applied activity (incubation at 37 °C for 1, 2, 4 and 6 h, RPMI 1640, 5 mm L-Gln, 5 mL non-essential amino acids (100×), 10% FCS + 5% BSA (*v*/*v*), 3.0 × 10^5^ cells/mL/well). (**b**) CCK-2R-mediated internalization of [^177^Lu]Lu-DOTA-rhCCK-16 (green), [^177^Lu]Lu-(*R*)-DOTAGA-rhCCK-16 (orange), [^177^Lu]Lu-DOTA-rhCCK-18 (red) and [^177^Lu]Lu-DOTA-rhCCK-16 (violet) after incubation at 37 °C for 6 h as percent of reference (% of [^177^Lu]Lu-DOTA-PP-F11N). * data taken from Holzleitner et al. [19]. These data have been determined in our lab under identical conditions.

For the ^177^Lu-labeled compounds rhCCK-16 and -18 containing either DOTA or (*R*)-DOTAGA as chelator, internalization studies were performed at different time points and compared to [^177^Lu]Lu-DOTA-PP-F11N (Figure 3, Table S2).

In general, all compounds demonstrated increasing internalization values on AR42J cells over time, while [^177^Lu]Lu-DOTA-rhCCK-16 and -18 exhibited a significantly higher internalization than their (*R*)-DOTAGA analogs and [^177^Lu]Lu-DOTA-PP-F11N at all time points (*p* < 0.001).

Stability studies on [^67^Ga]Ga-DOTA-rhCCK-16 and -18 as well as [^177^Lu]Lu-DOTA-rhCCK-16 and -18 in human serum (incubation at 37 °C for 24 h) revealed two major signals (Δ*t*_R_ ~ 2 min) for each derivative (Figure S18, Table S3). While the latter signal displays the amount of the respective intact compound (21–44%), the former was attributed to their analogs comprising a hydrolyzed SiFA moiety (SiOH-containing analog, 54–69%). The number of metabolites was <7% for all four derivatives.

### 3.3. In Vivo Characterization

**Figure 4:**
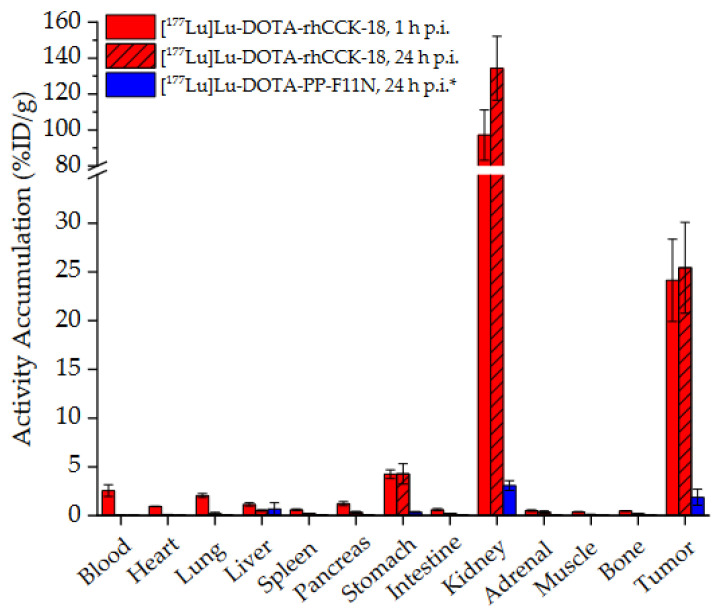
Biodistribution of [^177^Lu]Lu-DOTA-rhCCK-18 in selected organs (%ID/g) at 1 and 24 h p.i. in comparison to [^177^Lu]Lu-DOTA-PP-F11N at 24 h p.i. in AR42J tumor-bearing CB17-SCID mice (100 pmol each). Data is expressed as mean ± SD (*n* = 4). * data taken from Holzleitner et al. [19]. These data have been determined in our lab under identical conditions.

Due to its favorable in vitro data (highest CCK-2R affinity and internalization, excellent lipophilicity, preferable *HSA binding*), [^19^F]F-[^177^Lu]Lu-DOTA-rhCCK-18 was selected for further in vivo studies at 1 and 24 h p.i. (Figure 4, Table S4).

At 1 h p.i., activity levels of 24.1 ± 4.2 %ID/g were found for [^177^Lu]Lu-DOTA-rhCCK-18 in the AR42J tumor, which remained high over time, and levels of 25.4 ± 4.7 %ID/g were determined at 24 h p.i. (*p* > 0.35). Blood, heart and lung levels were slightly elevated at 1 h *p*.i. (0.9–2.6 %ID/g), while low levels (<0.2 %ID/g) were observed at 24 h p.i. in these organs (*p* < 0.01). Moreover, increased activity levels were found in the stomach at 1 and 24 h p.i., which was expected due to the endogenous CCK-2R expression in this organ. High kidney uptake was observed at 1 h p.i. for [^177^Lu]Lu-DOTA-rhCCK-18, which increased over time (*p* < 0.03). In comparison to the reference, [^177^Lu]Lu-DOTA-PP-F11N, [^177^Lu]Lu-DOTA-rhCCK-18 exhibited 13-fold increased tumor values at 24 h p.i. (*p* = 0.0001), while kidney values were also 40-fold enhanced (*p* < 0.0001).

**Figure 5:**
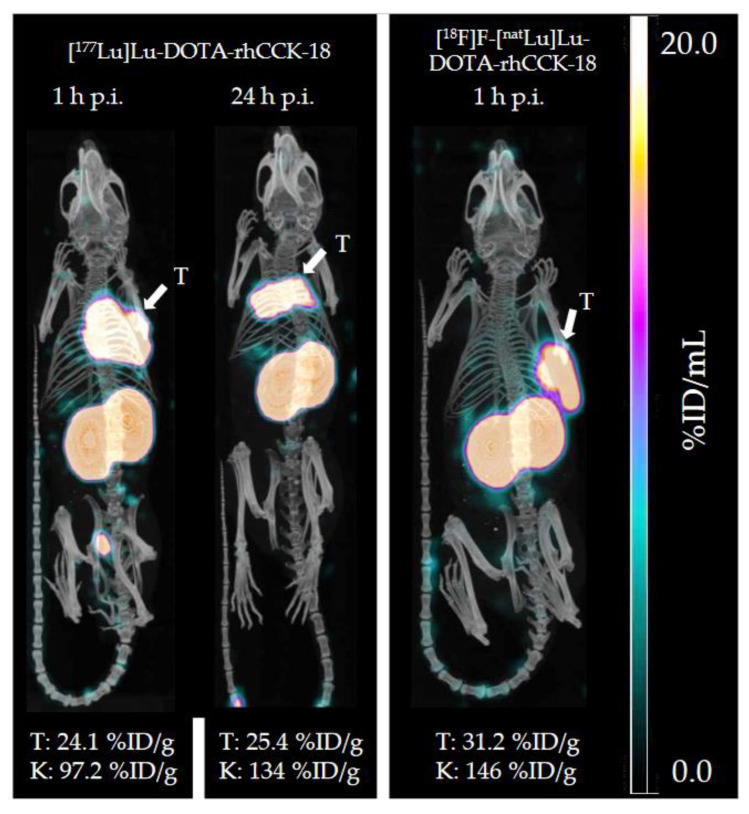
Representative *µ*SPECT/CT images of [^177^Lu]Lu-DOTA-rhCCK-18 at 1 and 24 h p.i. (**left**) and *µ*PET/CT image of [^18^F]F-[^nat^Lu]Lu-DOTA-rhCCK-18 at 1 h p.i. (**right**) in AR42J tumor-bearing CB17- SCID mice (100 pmol each). Tumors (T) are indicated by white arrows. Kidney (K) and tumor (T) values are depicted at the bottom.

*µ*SPECT/CT studies with [^177^Lu]Lu-DOTA-rhCCK-18 at 1 and 24 h p.i. revealed a low overall background activity at both time points, except for elevated tumor and kidney values (Figure 5, left). Moreover, the chemically identical compound, [^18^F]F-[^nat^Lu]Lu-DOTA-rhCCK-18, was evaluated via *µ*PET/CT in a AR42J tumor-bearing mouse (*n* = 1), which confirmed high tumor and kidney uptake at 1 h p.i. and low overall off-target accumulation (Figure 5, right).

The specificity of the uptake of [^177^Lu]Lu-DOTA-rhCCK-18 was confirmed via co-injection of excess (2.90 mg/kg, 40 nmol) of [^nat^Lu]Lu-DOTA-MGS5 (Figure S19).

## 4. Discussion

Due to its effective trapping via decarboxylation by the aromatic L-amino acid decarboxylase (AADC), [^18^F]F-DOPA is a clinically established neuroimaging agent, but can also be used for the detection of neuroendocrine tumors such as medullary thyroid carcinoma (MTC). Although high sensitivities are only observed for the detection of primary MTC lesions, [^18^F]F-DOPA is still considered the gold standard for MTC imaging in clinical practice [10,24], most likely as a consequence of the favorable properties of ^18^F-based positron emission tomography (PET) and the lack of reliable alternatives. Notwithstanding that the majority of MTC cells overexpress the cholecystokinin-2 receptor (CCK-2R) in high density, there is currently no CCK-2R-targeted compound available that shows promising pharmacokinetics and bears the possibility of ^18^F-labeling.

In recent studies, we thus introduced a silicon-based fluoride acceptor (SiFA) moiety into the D-glutamate chain of DOTA-PP-F11N. The resulting radiohybrid (rh)-based compounds enable labeling with both fluorine-18 and radiometals such as lutetium-177 due to the presence of a chelator and a SiFA moiety. We could show that these rhCCK ligands, for example [^nat/177^Lu]Lu-(*R*)-DOTAGA-rhCCK-16, revealed up to 8-fold increased activity levels in the tumor but also approximately 30-fold higher levels in the kidney when compared to [^nat/177^Lu]Lu-DOTA-PP-F11N, despite its distinctly lower CCK-2R affinity [19]. While we assume that the elevated tumor uptake and retention is due to a decelerated clearance of the compound, the charge distribution within the linker section and thus in proximity to the SiFA moiety likely causes the increased kidney values. Hence, the aim of this study was to retain favorable tumor values while reducing the activity levels in the kidneys. Therefore, (*R*)-DOTAGA was substituted by a DOTA chelator in two rhCCK derivatives to reduce the negative charges within the linker section and maintain the peptide sequence to retain high CCK-2R affinity.

Interestingly, substitution of (*R*)-DOTAGA by DOTA in the most affine rhCCK derivatives from previous studies, [^nat^Lu]Lu-(*R*)-DOTAGA-rhCCK-16 and -18, resulted in 3- to 4-fold lower *IC*_50_ values for the DOTA-comprising analogs, surpassing even the highly affine reference compound, [^nat^Lu]Lu-DOTA-PP-F11N (Figure 2). It is thus anticipated that the additional free carboxylic group of the (*R*)-DOTAGA chelator at the respective site has a negative impact on the overall CCK-2R affinity. Similar observations were made for the ^nat^Ga-labeled rhCCK ligands because the additional free carboxylic group at the Ga-(*R*)-DOTAGA chelate compared to the respective Ga-DOTA chelate as well as the additional free carboxylic group of the Ga-DOTA compared to the respective Lu-DOTA chelate [25,26,27] led to a decreased overall CCK-2R affinity (Figure 2). Stability studies in human serum did not reveal a lower stability for the [^67^Ga]Ga-DOTA-rhCCK-16 or -18 as compared to their ^177^Lu-labeled analogs, which can be thus excluded as a potential reason for the decreased CCK-2R affinity of the ^nat^Ga-labeled compounds. Interestingly, the stability studies in human serum showed the formation of a slightly more hydrophilic analog (Δ*t*_R_ ~ 2 min) for all four compounds tested (Figure S18). This was attributed to their corresponding SiOH-containing derivatives, and we suggest that the SiFA building block is hydrolyzed over time under these conditions. In order to confirm this assumption, we performed RP-HPLC analysis of the ^nat^Ga/^nat^Lu-labeled SiOH-containing analogs, which were generated by treatment with sodium hydroxide. All four SiOH-containing analogs (peptide identity confirmed by ESI-MS) revealed the same retention time as their respective ^67^Ga/^177^Lu-labeled derivative, which was observed after incubation in human serum. Because the SiFA- and their respective SiOH-containing ligands only differ by the atom/group bound to the silicon atom, but the remaining compound is identical, we do not consider this a metabolite but rather an intact compound. Further studies have to be conducted to elucidate whether the SiFA moiety is also hydrolyzed in vivo.

In addition to a lower CCK-2R affinity, all ^177^Lu-labeled (*R*)-DOTAGA-comprising compounds showed higher log*D*_7.4_ values than their DOTA-containing analogs (Table 1). Because it was assumed that the negatively charged ^177^Lu-(*R*)-DOTAGA chelates should be more hydrophilic than the neutral ^177^Lu-DOTA chelates, further investigations are necessary to understand this dedicated structure–activity relationship. Furthermore, the increased CCK-2R affinity was paralleled by an improved receptor-mediated internalization because both [^177^Lu]Lu-DOTA-rhCCK-16 and -18 exhibited the highest internalization values at all time points. While the slope of the internalization curves of most compounds decreases after the first hours (Figure 3a), the curve of [^177^Lu]Lu-DOTA-rhCCK-18 seems to rise with an almost unaffected slope up to the end of the experiment at 6 h. Thus, although this compound initially shows a decelerated internalization rate, its continuous cellular uptake might result in a noticeably higher overall uptake at later time points when compared with the other ligands of this series. Worth mentioning, both [^177^Lu]Lu-(*R*)-DOTAGA-rhCCK-16 and -18 showed significantly higher internalization values at all time points than [^177^Lu]Lu-DOTA-PP-F11N despite their significantly lower CCK-2R affinity, which points to a beneficial impact of the SiFA moiety on internalization kinetics.

In vivo, [^nat/177^Lu]Lu-DOTA-rhCCK-18 revealed a 1.5 and 13-fold increased activity uptake in the tumor (25.4 ± 4.7 %ID/g, Figure 4) at 24 h p.i., as compared to the previously published compound, [^177^Lu]Lu-(*R*)-DOTAGA-rhCCK-16 (15.7 ± 3.3 %ID/g), and the parent peptide, [^177^Lu]Lu-DOTA-PP-F11N (1.9 ± 0.8 %ID/g), respectively, which can be attributed to its significantly higher CCK-2R affinity and internalization [19]. Consequently, tumor-to-background ratios were higher for [^177^Lu]Lu-DOTA-rhCCK-18 compared to the previously published compounds (Table S5). Tumor specificity was demonstrated by competition studies using excess of the CCK-2R-specific compound, [^nat^Lu]Lu-DOTA-MGS5 [28], which led to tumor and stomach values <2 %ID/g. Moreover, the high tumor values obtained for [^177^Lu]Lu-DOTA-rhCCK-18 demonstrated that the low tumor values for [^177^Lu]Lu-DOTA-PP-F11N were not caused by an excessive amount of substance (100 pmol per animal) for such a high-affinity ligand.

Similar to previously published rhCCK derivatives [19], tumor and kidney uptake for [^177^Lu]Lu-DOTA-rhCCK-18 was high at 1 h p.i. and remained high at 24 h p.i. Elevated tumor and stomach retention can be attributed to the decelerated clearance kinetics (higher log*D*_7.4_ and albumin binding) of the rhCCK ligands and their prolonged bioavailability, the increased kidney retention is likely caused by a synergistic effect of the negatively charged side chains in proximity of the SiFA building block. This assumption is supported by the fact that [^177^Lu]Lu-DOTA-PP-F11N contains a similar number of negative charges but no SiFA moiety and does not show an enhanced kidney retention. Recent reports demonstrated that SiFA-comprising PSMA inhibitors show a higher albumin binding and thus decelerated clearance kinetics, which results in increased tumor uptake, but also increased kidney retention [21,29], which correlated well with our observations because [^177^Lu]Lu-DOTA-rhCCK-18 also exhibited an elevated albumin binding in vitro. Furthermore, it was shown that negative charges in the direct neighborhood to the SiFA moiety cause a higher albumin binding and stronger kidney retention, which could explain our results because the rhCCK derivatives contain several negative charges in proximity to the SiFA group. As the substitution of a (*R*)-DOTAGA by a DOTA moiety did not result in lower kidney retention, it is assumed that most of the negatively charged *γ*-D-glutamic acid moieties have to be removed in future studies to prevent an elevated kidney uptake and retention. In addition, it will be interesting to see whether the impact of these negative charges of radiohybrid and other CCK-2R ligands on the kidney retention will be confirmed by the first comparative studies in humans.

Nevertheless, even in the case that such behavior would be confirmed in human studies, unfavorable kidney uptake of [^19^F]F-[^177^Lu]Lu-DOTA-rhCCK-18 does not necessarily prevent its use for PET imaging with the corresponding ^18^F-radiohybrid. When ^18^F-labeled, and taking into account the short half-life of fluorine-18, an elevated kidney accumulation will not result in an unacceptable dosimetry. Similar kidney uptake is, for example, also observed for commonly applied PSMA inhibitors [30,31,32,33]. Despite this minor disadvantage, [^18^F]F-[^nat^Lu]Lu-DOTA-rhCCK-18 seems to have great potential for the detection of even small and distant metastases in MTC patients due to the unique properties of ^18^F-PET and the high overexpression of the CCK-2R on these cancer cells.

In order to confirm the expected favorable pharmacokinetics of the chemically identical [^18^F]F-[^nat^Lu]Lu-DOTA-rhCCK-18, we carried out a *µ*PET/CT image (*n* = 1, Figure 5), which revealed similarly high activity levels in the tumor and kidneys compared to [^19^F]F-[^177^Lu]Lu-DOTA-rhCCK-18. Moreover, bone uptake was observed to be low (1.69 %ID/g) for [^18^F]F-[^nat^Lu]Lu-DOTA-rhCCK-18, underlining the high metabolic stability of the Si-^18^F bond. Therefore, the formation of the SiOH-containing analog observed in stability studies in human serum does not seem to occur in vivo, at least not within the first hours after injection, and should therefore not be of concern for PET imaging using this compound. However, stability of this compound (particularly of the Si-F bond) in men must be investigated to confirm this assumption. Due to these results, particularly its high tumor accumulation at 1 h p.i., [^18^F]F-[^nat^Lu]Lu-DOTA-rhCCK-18 could surpass the detection rate of currently applied CCK-2R-targeted compounds such as [^111^In]In-CP04 or [^68^Ga]Ga-DOTA-MGS5 [9,28,34,35] and might even compete with the current gold standard for MTC imaging, [^18^F]F-DOPA, i.e., for the detection of distant metastases.

^18^F-labeling was carried out via an isotopic exchange reaction by a novel labeling strategy, which led to molar activities of ~85 GBq/µmol in a total synthesis time of ~30 min. Unlike the Munich Method [36], this strategy includes the use of ammonium formiate (in anhydrous DMSO) instead of [K^+^⊂2.2.2]OH^−^ (in anhydrous MeCN) for the elution of dry [^18^F]fluoride from a SEP-Pak^®^ Light (46 mg) Accell Plus QMA cartridge (Waters GmbH, Eschborn, Germany), which enables a less time-consuming preparation and more cost efficient ^18^F-fluorination method. In comparison to conventional radiofluorination techniques [37], no azeotropic drying steps must be conducted. Furthermore, anhydrous DMSO is used as the reaction solvent, which is beneficial for the ^18^F-labeling of CCK-2R-targeting peptides.

In summary, we could demonstrate a significantly higher CCK-2R affinity and thus enhanced tumor accumulation by exchanging the chelator moiety in previously published rhCCK derivatives from (*R*)-DOTAGA to DOTA. Nevertheless, elevated kidney retention of rhCCK derivatives could not be reduced in this study, which must be addressed in future studies by extinguishing most of the negatively charged residues within the SiFA-containing linker section. Despite increased kidney uptake, [^18^F]F-[^nat^Lu]Lu-DOTA-rhCCK-18 holds great promise as an imaging agent and is expected to be highly competitive to currently applied radiotracers for PET imaging of medullary thyroid carcinoma.

## 5. Conclusions

While a simple substitution of (*R*)-DOTAGA by DOTA in previously reported rhCCK derivatives led to a noticeably increased CCK-2R affinity and thus high activity levels in the tumor for [^19^F]F-[^177^Lu]Lu-DOTA-rhCCK-18 at 24 h p.i., kidney retention was also high. Nevertheless, due to its very high tumor accumulation at already 1 h p.i., the chemically identical [^18^F]F-[^nat^Lu]Lu-DOTA-rhCCK-18 might compete with or even surpass the detection rate of currently applied imaging agents for MTC such as ^68^Ga- or ^111^In-labeled CCK-2R or SSTR2-targeted compounds and [^18^F]F-DOPA, which is why a clinical translation of this compound for MTC imaging is recommended.

## Figures and Tables

**Table 1:** Lipophilicity (log*D*_7.4_) and HSA binding data of the ^177^Lu-labeled peptides.

Compound	Log*d*_7.4_	HSA Binding (%)
[^177^Lu]Lu-DOTA-PP-F11N	−4.75 ± 0.07	n.d.
[^177^Lu]Lu-(*R*)-DOTAGA-PP-F11N	−3.95 ± 0.06	n.d.
[^177^Lu]Lu-DOTA-rhCCK-16	−2.70 ± 0.09	89.1%
[^177^Lu]Lu-(*R*)-DOTAGA-rhCCK-16 *	−2.54 ± 0.05	n.d.
[^19^F]F-[^177^Lu]Lu-DOTA-rhCCK-18	−2.69 ± 0.06	62.6%
[^18^F]F-[^nat^Lu]Lu-DOTA-rhCCK-18	−2.71 ± 0.04	n.d.
[^177^Lu]Lu-(*R*)-DOTAGA-rhCCK-18 *	−2.16 ± 0.09	n.d.

* data taken from Holzleitner et al. [19]. These data have been determined in our lab under identical conditions, n.d.: not determined.

## Data Availability

Data is contained within the article and Supplementary Materials.

## Supplementary Materials

The following supporting information can be downloaded at: https://www.mdpi.com/article/10.3390/pharmaceutics15030826/s1, general information and characterization of all CCK2R-targeted compounds, detailed description of cell-based experiments. Figure S1: (a) Confirmation of peptide identity and integrity for [^nat^Lu]Lu-DOTA-PP-F11N, as analyzed by analytical RP-HPLC (10→90% MeCN in H_2_O + 0.1% TFA in 15 min). (b) Mass spectrum of [^nat^Lu]Lu-DOTA-PP-F11N. (c) Confirmation of peptide identity and integrity for [^177^Lu]Lu-DOTA-PP-F11N, as analyzed by analytical (radio-)RP-HPLC (10→90% MeCN in H_2_O + 0.1% TFA in 15 min); Figure S2: (a) Confirmation of peptide identity and integrity for [^nat^Lu]Lu-(*R*)-DOTAGA-PP-F11N, as analyzed by analytical RP-HPLC (10→90% MeCN in H_2_O + 0.1% TFA in 15 min). (b) Mass spectrum of [^nat^Lu]Lu-(*R*)-DOTAGA-PP-F11N. (c) Confirmation of peptide identity and integrity for [^177^Lu]Lu-(*R*)-DOTAGA-PP-F11N, as analyzed by analytical (radio-)RP-HPLC (10→90% MeCN in H_2_O + 0.1% TFA in 15 min); Figure S3: (a) Confirmation of peptide identity and integrity for [^nat^Lu]Lu-DOTA-rhCCK-16, as analyzed by analytical RP-HPLC (10→90% MeCN in H_2_O + 0.1% TFA in 15 min). (b) Mass spectrum of [^nat^Lu]Lu-DOTA-rhCCK-16. (c) Confirmation of peptide identity and integrity for [^177^Lu]Lu-DOTA-rhCCK-16, as analyzed by analytical (radio-)RP-HPLC (10→90% MeCN in H_2_O + 0.1% TFA in 15 min); Figure S4: (a) Confirmation of peptide identity and integrity for [^177^Lu]Lu-DOTA-rhCCK-16.2, as analyzed by analytical RP-HPLC (10→30% MeCN in H_2_O + 0.1% TFA in 5 min, 30→60% MeCN in H_2_O + 0.1% TFA in 15 min). (b) Mass spectrum of [^nat^Lu]Lu-DOTA-rhCCK-16.2; Figure S5: (a) Confirmation of peptide identity and integrity for [^nat^Lu]Lu-(*R*)-DOTAGA-rhCCK-16, as analyzed by analytical RP-HPLC (10→90% MeCN in H_2_O + 0.1% TFA in 15 min). (b) Mass spectrum of [^nat^Lu]Lu-(*R*)-DOTAGA-rhCCK-16. (c) Confirmation of peptide identity and integrity for [^177^Lu]Lu-(*R*)-DOTAGA-rhCCK-16, as analyzed by analytical (radio-)RP-HPLC (10→90% MeCN in H_2_O + 0.1% TFA in 15 min); Figure S6: (a) Confirmation of peptide identity and integrity for [^nat^Lu]Lu-DOTA-rhCCK-18, as analyzed by analytical RP-HPLC (10→90% MeCN in H_2_O + 0.1% TFA in 15 min). (b) Mass spectrum of [^nat^Lu]Lu-DOTA-rhCCK-18. (c) Confirmation of peptide identity and integrity for [^177^Lu]Lu-DOTA-rhCCK-18, as analyzed by analytical (radio-)RP-HPLC (10→90% MeCN in H_2_O + 0.1% TFA in 15 min); Figure S7: Confirmation of peptide identity and integrity for [^18^F]F-[^nat^Lu]Lu-DOTA-rhCCK-18, as analyzed by analytical radio-RP-HPLC (10→70% MeCN in H_2_O + 0.1% TFA in 15 min); Figure S8: (a) Confirmation of peptide identity and integrity for [^177^Lu]Lu-DOTA-rhCCK-18.2, as analyzed by analytical RP-HPLC (10→30% MeCN in H_2_O + 0.1% TFA in 5 min, 30→60% MeCN in H_2_O + 0.1% TFA in 15 min). (b) Mass spectrum of [^nat^Lu]Lu-DOTA-rhCCK-18.2; Figure S9: (a) Confirmation of peptide identity and integrity for [^nat^Lu]Lu-(*R*)-DOTAGA-rhCCK-18, as analyzed by analytical RP-HPLC (10→90% MeCN in H_2_O + 0.1% TFA in 15 min). (b) Mass spectrum of [^nat^Lu]Lu-(*R*)-DOTAGA-rhCCK-18. (c) Confirmation of peptide identity and integrity for [^177^Lu]Lu-(*R*)-DOTAGA-rhCCK-18, as analyzed by analytical (radio-)RP-HPLC (10→90% MeCN in H_2_O + 0.1% TFA in 15 min); Figure S10: (a) Confirmation of peptide identity and integrity for [^nat^Ga]Ga-(*R*)-DOTAGA-PP-F11N, as analyzed by analytical RP-HPLC (10→90% MeCN in H_2_O + 0.1% TFA in 15 min). (b) Mass spectrum of [^nat^Ga]Ga-(*R*)-DOTAGA-PP-F11N; Figure S11: (a) Confirmation of peptide identity and integrity for [^nat^Ga]Ga-DOTA-PP-F11N, as analyzed by analytical RP-HPLC (10→90% MeCN in H_2_O + 0.1% TFA in 15 min). (b) Mass spectrum of [^nat^Ga]Ga-DOTA-PP-F11N; Figure S12: (a) Confirmation of peptide identity and integrity for [^nat^Ga]Ga-DOTA-rhCCK-16, as analyzed by analytical RP-HPLC (10→90% MeCN in H_2_O + 0.1% TFA in 15 min). (b) Mass spectrum of [^nat^Ga]Ga-DOTA-rhCCK-16; Figure S13: (a) Confirmation of peptide identity and integrity for [^67^Ga]Ga-DOTA-rhCCK-16.2, as analyzed by analytical RP-HPLC (10→30% MeCN in H_2_O + 0.1% TFA in 5 min, 30→60% MeCN in H_2_O + 0.1% TFA in 15 min). (b) Mass spectrum of [^nat^Ga]Ga-DOTA-rhCCK-16.2; Figure S14: (a) Confirmation of peptide identity and integrity for [^nat^Ga]Ga-(*R*)-DOTAGA-rhCCK-16, as analyzed by analytical RP-HPLC (10→90% MeCN in H_2_O + 0.1% TFA in 15 min). (b) Mass spectrum of [^nat^Ga]Ga-(*R*)-DOTAGA-rhCCK-16; Figure S15: (a) Confirmation of peptide identity and integrity for [^nat^Ga]Ga-DOTA-rhCCK-18, as analyzed by analytical RP-HPLC (10→90% MeCN in H_2_O + 0.1% TFA in 15 min). (b) Mass spectrum of [^nat^Ga]Ga-DOTA-rhCCK-18; Figure S16: (a) Confirmation of peptide identity and integrity for [^67^Ga]Ga-DOTA-rhCCK-18.2, as analyzed by analytical RP-HPLC (10→30% MeCN in H_2_O + 0.1% TFA in 5 min, 30→60% MeCN in H_2_O + 0.1% TFA in 5 min). (b) Mass spectrum of [^nat^Ga]Ga-DOTA-rhCCK-18.2; Figure S17: (a) Confirmation of peptide identity and integrity for [^nat^Ga]Ga-(*R*)-DOTAGA-rhCCK-18, as analyzed by analytical RP-HPLC (10→90% MeCN in H_2_O + 0.1% TFA in 15 min). (b) Mass spectrum of [^nat^Ga]Ga-(*R*)-DOTAGA-rhCCK-18; Figure S18: Stability studies of (a) [^177^Lu]Lu-DOTA-rhCCK-16, (b) [^177^Lu]Lu-DOTA-rhCCK-18, (c) [^67^Ga]Ga-DOTA-rhCCK-16, and (d) [^67^Ga]Ga-DOTA-rhCCK-18 in human serum (37 °C, 24 h), as analyzed by analytical RP-HPLC (10→30% MeCN in H_2_O + 0.1% TFA in 5 min, 30→60% MeCN in H_2_O + 0.1% TFA in 5 min). The chromatograms of the respective compounds after incubation in human serum (37 °C, 24 h) are depicted in red. Quality controls of the intact compounds comprising a SiFA moiety are depicted in gray and quality controls of the SiOH-comprising analogs (“hydrolyzed SiFA moiety”) are depicted in blue; Figure S19: (a) Biodistribution and (b) a representative *µ*SPECT/CT image of [^177^Lu]Lu-DOTA-rhCCK-18 (100 pmol) co-injected with [^nat^Lu]Lu-DOTA-MGS5 (40 nmol) in selected organs (%ID/g) at 24 h p.i. in AR42J tumor-bearing CB17-SCID mice. Data is expressed as mean ± SD (*n =* 2); Table S1: Affinity data (*n* = 3) of the compounds evaluated, determined on AR42J cells (2.0 × 10^5^ cells/well) with [^177^Lu]Lu-DOTA-PP-F11N (0.3 pmol/well) as radiolabeled reference (3 h, 37 °C, RPMI 1640, 5 mM L-Gln, 5 mL non-essential amino acids (100×), 10% FCS + 5% BSA (*v*/*v*)); Table S2: Receptor-mediated internalization values (37 °C, RPMI 1640, 5 mM L-Gln, 5 mL non-essential amino acids (100×), 10% FCS, 0.25 pmol/well) determined as percentages (%) of the applied activity of [^177^Lu]Lu-(*R*)-DOTAGA-rhCCK-18 as well as [^177^Lu]Lu-DOTA-rhCCK-16 and -18 using AR42J cells (3.0 × 10^5^ cells/well) at different time points (1, 2, 4 and 6 h). Data are corrected for non-specific binding (10 µmol, [^nat^Lu]Lu-DOTA-PP-F11N); Table S3: Amounts of intact peptides and their analogs containing a hydrolyzed SiFA (=SiOH) moiety (*n* = 3) of the compounds evaluated, determined in human serum after incubation at 37 °C for 24 h; Table S4: Biodistribution data of [^177^Lu]Lu-DOTA-rhCCK-18 in selected organs at 1 and 24 h p.i. in AR42J tumor-bearing CB17-SCID mice (100 pmol each). Data are expressed as %ID/g, mean ± SD (*n* = 4). Biodistribution data of [^177^Lu]Lu-DOTA-rhCCK-18 (100 pmol) co-injected with [^177^Lu]Lu-DOTA-MGS5 in selected organs at 24 h p.i. in AR42J tumor-bearing CB17-SCID mice. Data are expressed as %ID/g, mean ± SD (*n* = 2); Table S5: Tumor-to-background ratios of [^177^Lu]Lu-DOTA-rhCCK-18, [^177^Lu]Lu-(*R*)-DOTAGA-rhCCK-16 and [^177^Lu]Lu-(*R*)-DOTAGA-rhCCK-9 for the selected organs of AR42J tumor-bearing CB17-SCID mice at 24 h p.i. (100 pmol each). Data are expressed as mean ± SD (*n* = 4).
