# Supplementary material for: Development of the First 18F-Labeled Radiohybrid-Based Minigastrin Derivative with High Target Affinity and Tumor Accumulation by Substitution of the Chelating Moiety
Source: Pharmaceutics. 2023 Mar 3;15(3):826. doi: 10.3390/pharmaceutics15030826 (PMC10054553; doi:10.3390/pharmaceutics15030826)
Supplement: Supplementary file 1 [file pharmaceutics-15-00826-s001.zip › pharmaceutics-2189665-supplementary.pdf]

Development of the First ^{18}F-Labeled Radiohybrid-Based Minigastrin Derivative with High Target Affinity and Tumor Accumulation by Substitution of the Chelating Moiety

# - Supplementary Materials -

Thomas Günther [1,*,†], Nadine Holzleitner [1,†], Daniel Di Carlo [1], Roswitha Beck [1], Constantin Lapa [2] and Hans-Jürgen Wester [1]

General information

Analytical and preparative reversed-phase high performance liquid chromatography (RP-HPLC) were performed using Shimadzu gradient systems (Shimadzu Deutschland GmbH, Neufahrn, Germany), each equipped with an SPD-20A UV/Vis detector (220 nm, 254 nm). Different gradients of MeCN (0.1% TFA, 2 or 5% H_2O for analytical or preparative applications, respectively) in H_2O (0.1% TFA) were used as eluents for all RP-HPLC operations.
For analytical measurements, a MultoKrom 100-5 C18 (150 mm x 4.6 mm) column (CS Chromatographie Service GmbH, Langerwehe, Germany) was used at a flow rate of 1 mL/min. Both specific gradients and the corresponding retention times t_R as well as the capacity factor K' are cited in the text.
Preparative RP-HPLC purification was performed using a MultoKrom 100-5 C18 (250 mm x 20 mm) column (CS Chromatographie GmbH, Langerwehe, Germany) at a constant flow rate of 10 mL/min.
Lyophilization was accomplished using an Alpha 1-2 LDplus lyophilizer (Martin Christ Gefriertrocknungsanlagen GmbH, Osterode am Harz, Deutschland) combined with a RZ-2 vacuum pump (Vacuubrand GmbH & Co KG, Olching, Germany).
Analytical and preparative radio RP-HPLC was performed using a MultoKrom 100-5 C18 (5 µm, 125 × 4.6 mm) column (CS Chromatographie GmbH, Langerwehe, Germany). A HERM LB 500 NaI scintillation detector (Berthold Technologies, Bad Wildbad, Germany) was connected to the outlet of the UV photometer for the detection of radioactivity.
Radioactive samples were measured by a WIZARD2® 2480 Automatic γ-Counter (Perkin Elmer Inc., Waltham, MA, USA).

Analytical data of nat/177Lu-labeled minigastrin analogs

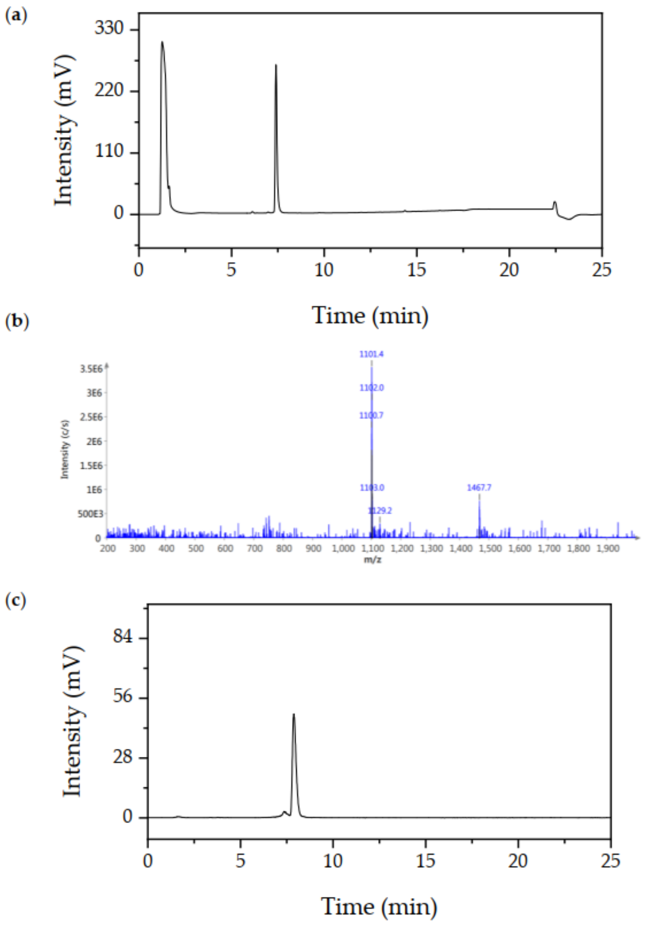

Figure S1. (**a**) Confirmation of peptide identity and integrity for [natLu]Lu-DOTA-PP-F11N, as analyzed by analytical RP-HPLC (10→90% MeCN in H_2O + 0.1% TFA in 15 min). (**b**) Mass spectrum of [natLu]Lu-DOTA-PP-F11N. (**c**) Confirmation of peptide identity and integrity for [177Lu]Lu-DOTA-PP-F11N, as analyzed by analytical (radio-)RP-HPLC (10→90% MeCN in H_2O + 0.1% TFA in 15 min).

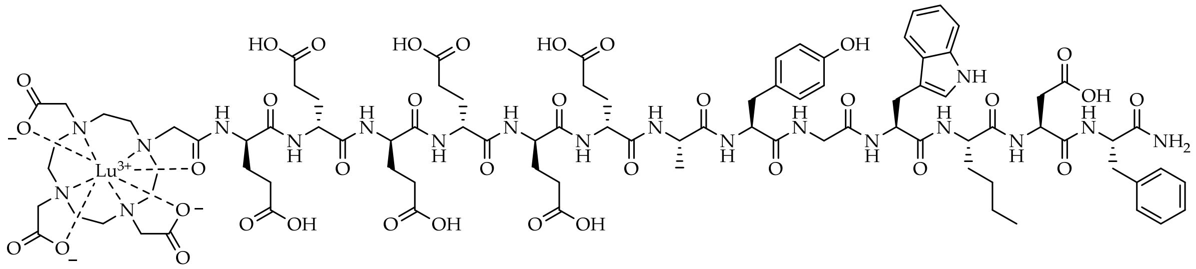

[natLu]Lu-DOTA-PP-F11N: RP-HPLC (10→90% MeCN in H_2O with 0.1% TFA, 15 min, $\lambda = 220$ nm): $t_R = 7.55$ min, K′ = 3.48; MS (ESI, positive): m/z calculated for $C_{90}H_{120}LuN_{19}O_{35}$: 2203.0, found: m/z = 1101.4 $[M+2H]^{2+}$.

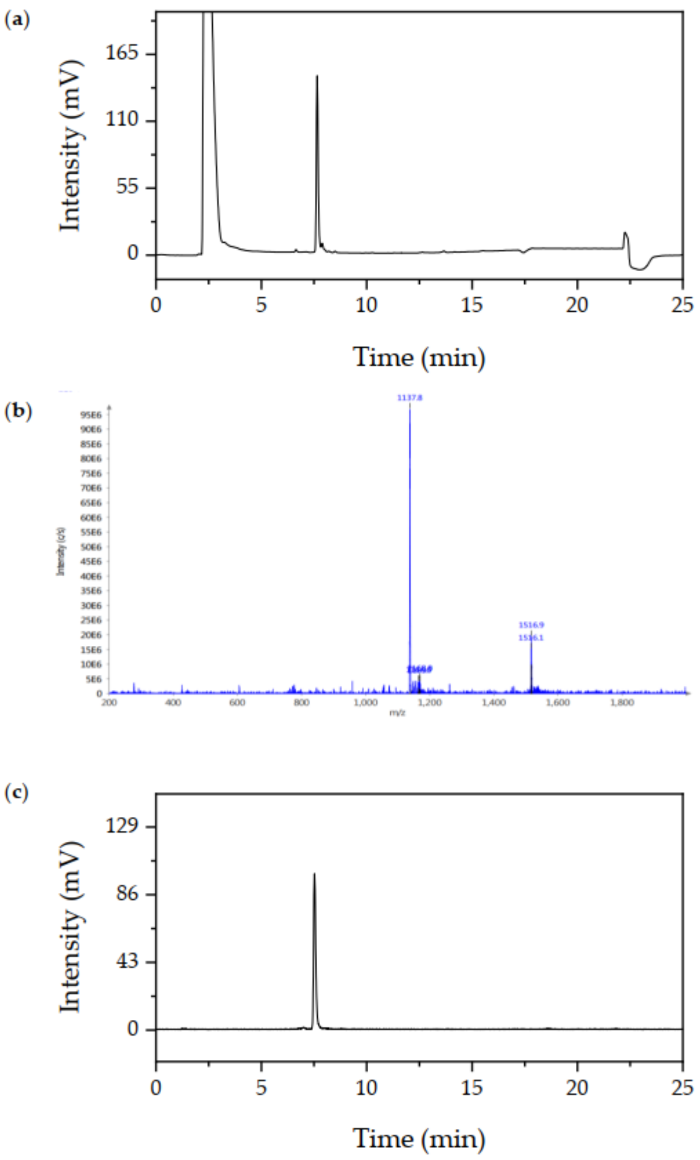

Figure S2. (**a**) Confirmation of peptide identity and integrity for [natLu]Lu-(*R*)-DOTAGA-PP-F11N, as analyzed by analytical RP-HPLC (10→90% MeCN in H_2O + 0.1% TFA in 15 min). (**b**) Mass spectrum of [natLu]Lu-(*R*)-DOTAGA-PP-F11N. (**c**) Confirmation of peptide identity and integrity for [177Lu]Lu-(*R*)-DOTAGA-PP-F11N, as analyzed by analytical (radio-)RP-HPLC (10→90% MeCN in H_2O + 0.1% TFA in 15 min).

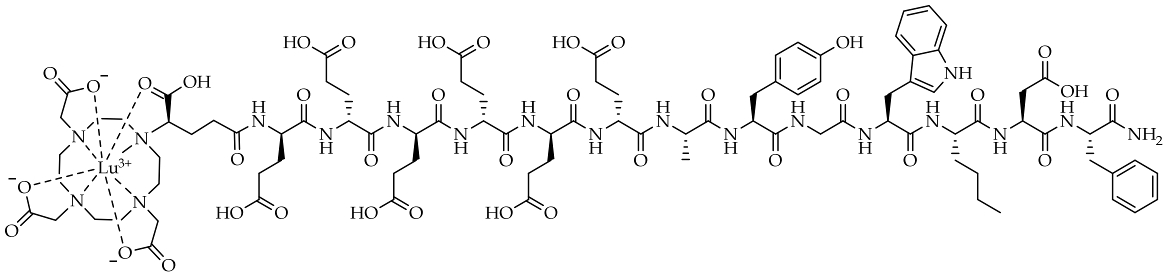

[natLu]Lu-(R)-DOTAGA-PP-F11N: RP-HPLC (10→90% MeCN in H_2O with 0.1% TFA, 15 min, λ = 220 nm): t_R = 7.8 min, K′ = 3.88; MS (ESI, positive): m/z calculated for $C_{93}H_{124}LuN_{19}O_{37}$: 2273.8, found: m/z = 1137.8 $[M+2H]^{2+}$.

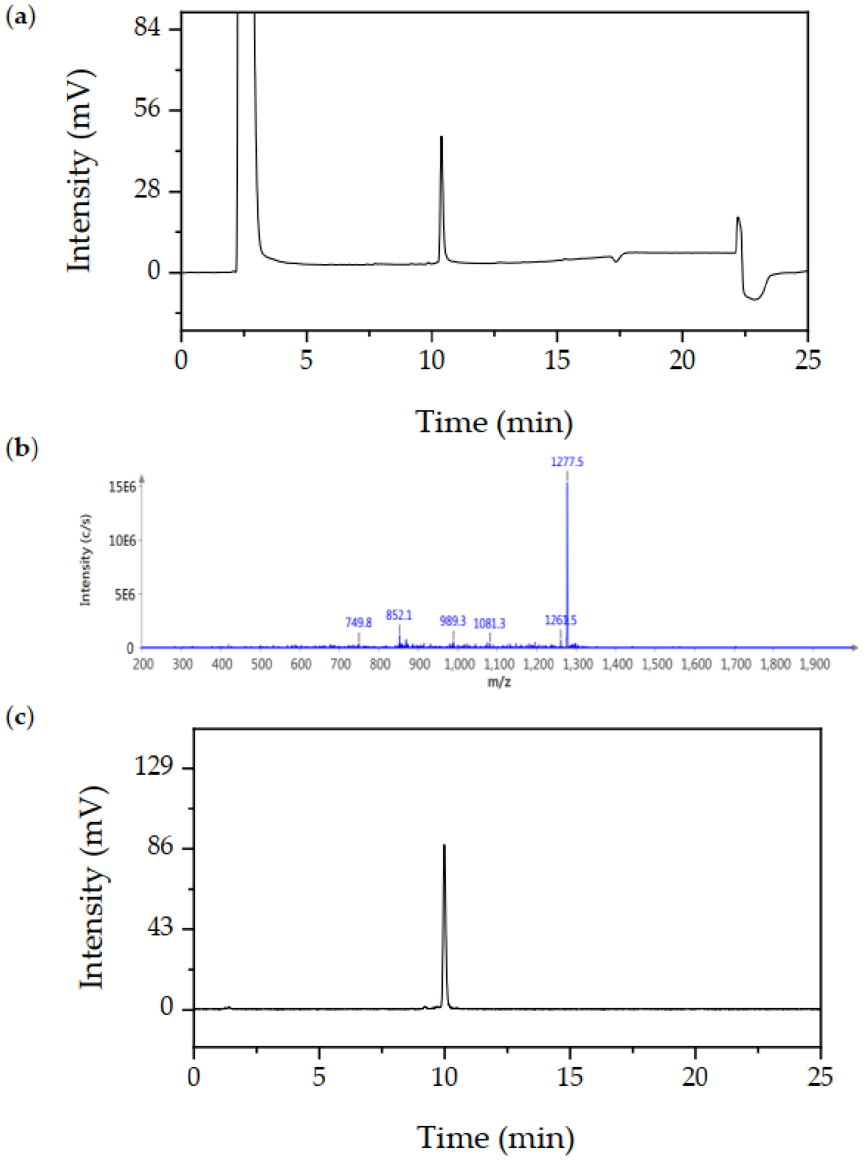

Figure S3. (**a**) Confirmation of peptide identity and integrity for [natLu]Lu-DOTA-rhCCK-16, as analyzed by analytical RP-HPLC (10→90% MeCN in H_2O + 0.1% TFA in 15 min). (**b**) Mass spectrum of [natLu]Lu-DOTA-rhCCK-16. (**c**) Confirmation of peptide identity and integrity for [177Lu]Lu-DOTA-rhCCK-16, as analyzed by analytical (radio-)RP-HPLC (10→90% MeCN in H_2O + 0.1% TFA in 15 min).

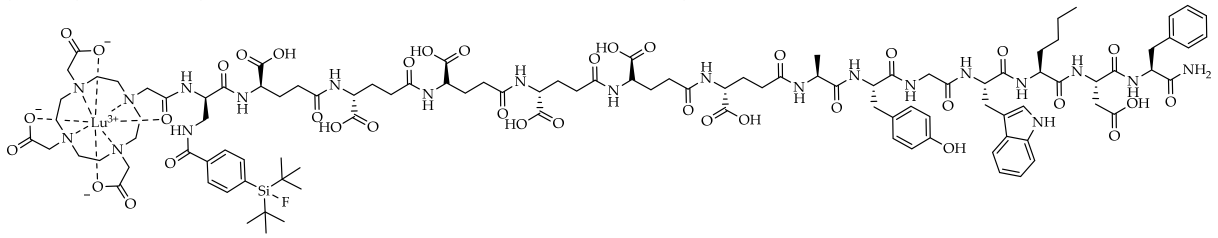

[natLu]Lu-DOTA-rhCCK-16: RP-HPLC (10→90% MeCN in H_2O with 0.1% TFA, 15 min, λ = 220 nm): t_R = 10.4 min, K′ = 5.16; MS (ESI, positive): m/z calculated for $C_{108}H_{147}FLuN_{21}O_{37}Si$: 2553.5, found: m/z = 1277.5 $[M+2H]^{2+}$.

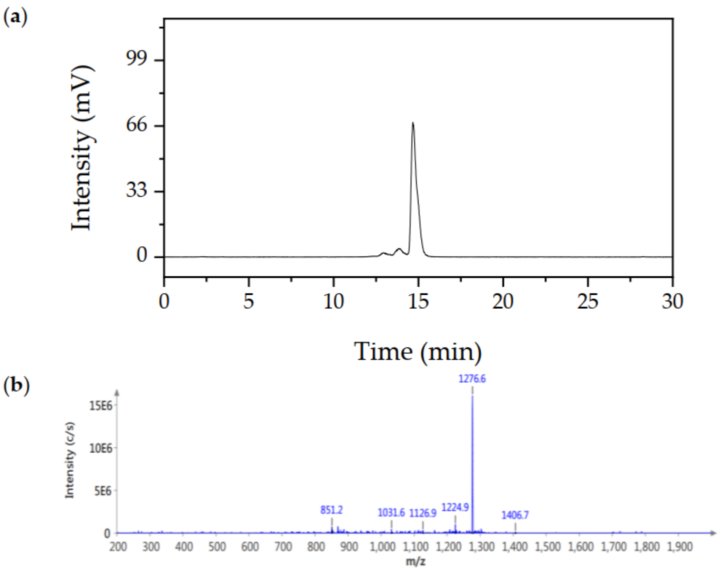

Figure S4. (**a**) Confirmation of peptide identity and integrity for [^{177}Lu]Lu-DOTA-rhCCK-16.2, as analyzed by analytical RP-HPLC (10→30% MeCN in H_2O + 0.1% TFA in 5 min, 30→60% MeCN in H_2O + 0.1% TFA in 15 min). (**b**) Mass spectrum of [^{nat}Lu]Lu-DOTA-rhCCK-16.2.

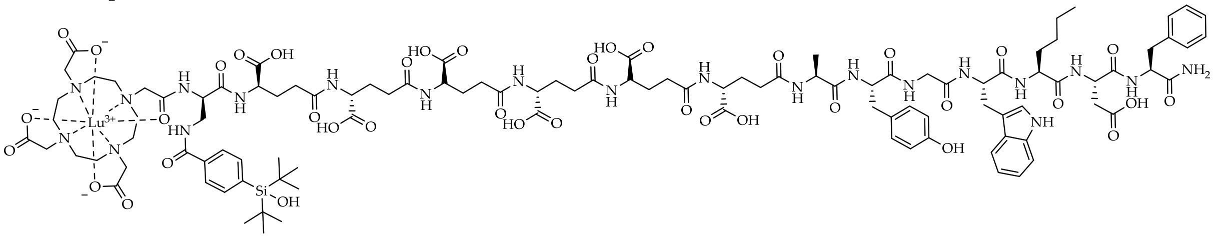

[$^{nat/177}$Lu]Lu-DOTA-rhCCK-16.2: RP-HPLC (10→30% MeCN in H_2O with 0.1% TFA, 5 min, 30→60% MeCN in H_2O with 0.1% TFA, 15 min, λ = 220 nm): t_R = 14.7 min, K' = 6.35; MS (ESI, positive): m/z calculated for $C_{108}H_{148}LuN_{21}O_{38}Si$: 2551.5, found: m/z = 1276.6 $[M+2H]^{2+}$.

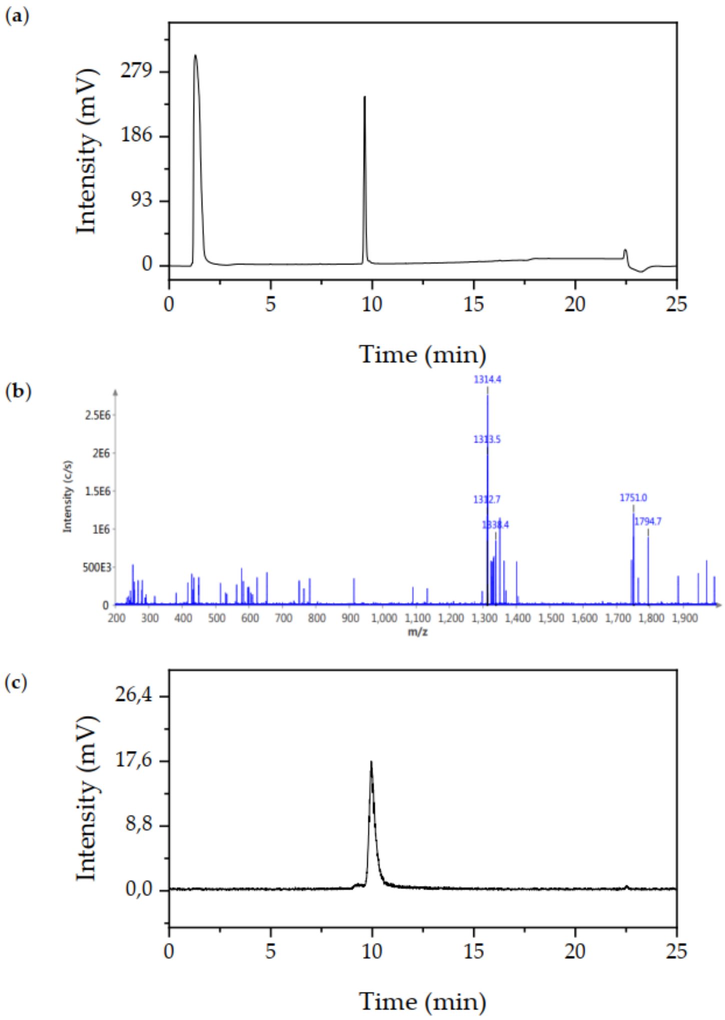

Figure S5. (**a**) Confirmation of peptide identity and integrity for [natLu]Lu-(*R*)-DOTAGA-rhCCK-16, as analyzed by analytical RP-HPLC (10→90% MeCN in H_2O + 0.1% TFA in 15 min). (**b**) Mass spectrum of [natLu]Lu-(*R*)-DOTAGA -rhCCK-16. (**c**) Confirmation of peptide identity and integrity for [177Lu]Lu-(*R*)-DOTAGA-rhCCK-16, as analyzed by analytical (radio-)RP-HPLC (10→90% MeCN in H_2O + 0.1% TFA in 15 min).

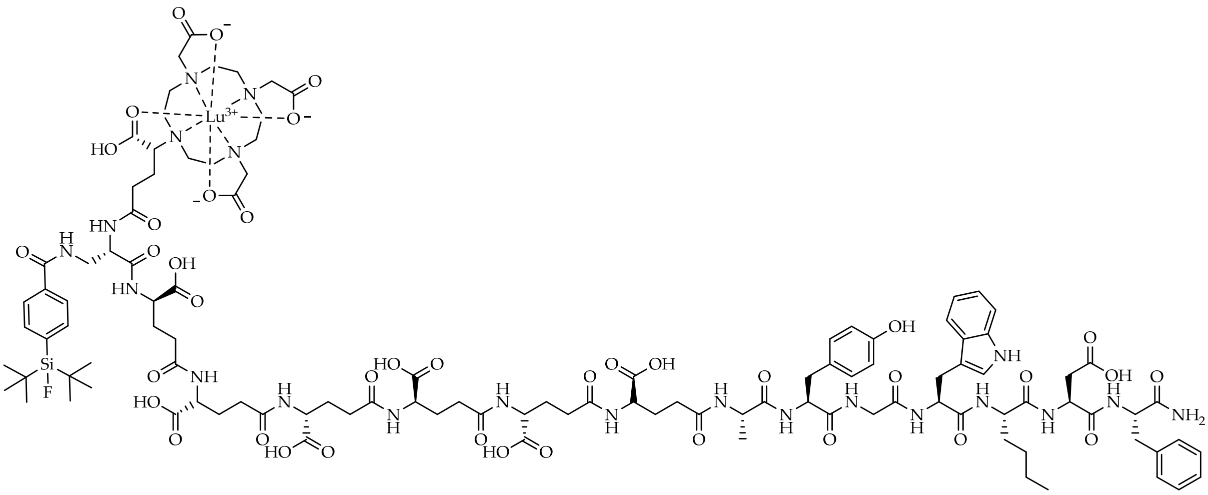

[natLu]Lu-(R)-DOTAGA-rhCCK-16: RP-HPLC (10 → 90% MeCN in H_2O with 0.1% TFA, 15 min, λ = 220 nm): t_R = 9.52 min, K′ = 4.64; MS (ESI, positive): m/z calculated for $C_{111}H_{151}FLuN_{21}O_{39}Si$: 2625.6, found: m/z = 1314.4 $[M+2H]^{2+}$.

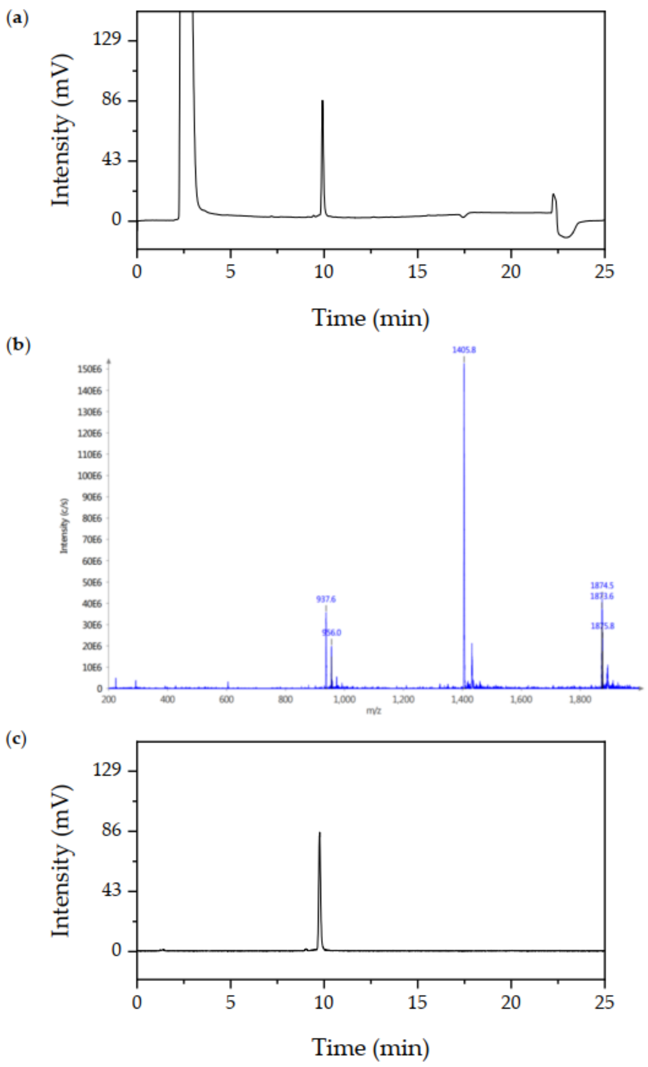

Figure S6. (**a**) Confirmation of peptide identity and integrity for [natLu]Lu-DOTA-rhCCK-18, as analyzed by analytical RP-HPLC (10→90% MeCN in H_2O + 0.1% TFA in 15 min). (**b**) Mass spectrum of [natLu]Lu-DOTA-rhCCK-18. (**c**) Confirmation of peptide identity and integrity for [177Lu]Lu-DOTA-rhCCK-18, as analyzed by analytical (radio-)RP-HPLC (10→90% MeCN in H_2O + 0.1% TFA in 15 min).

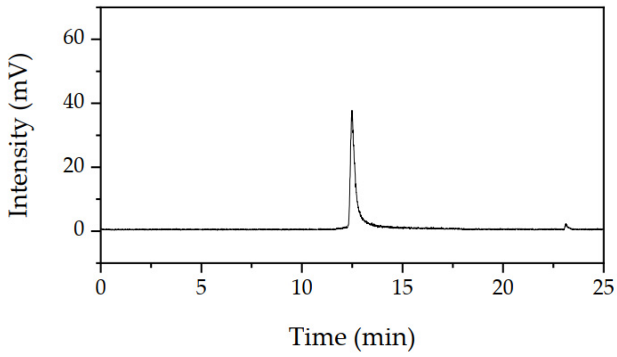

Figure S7. Confirmation of peptide identity and integrity for [18F]F-[natLu]Lu-DOTA-rhCCK-18, as analyzed by analytical radio-RP-HPLC (10→70% MeCN in H_2O + 0.1% TFA in 15 min).

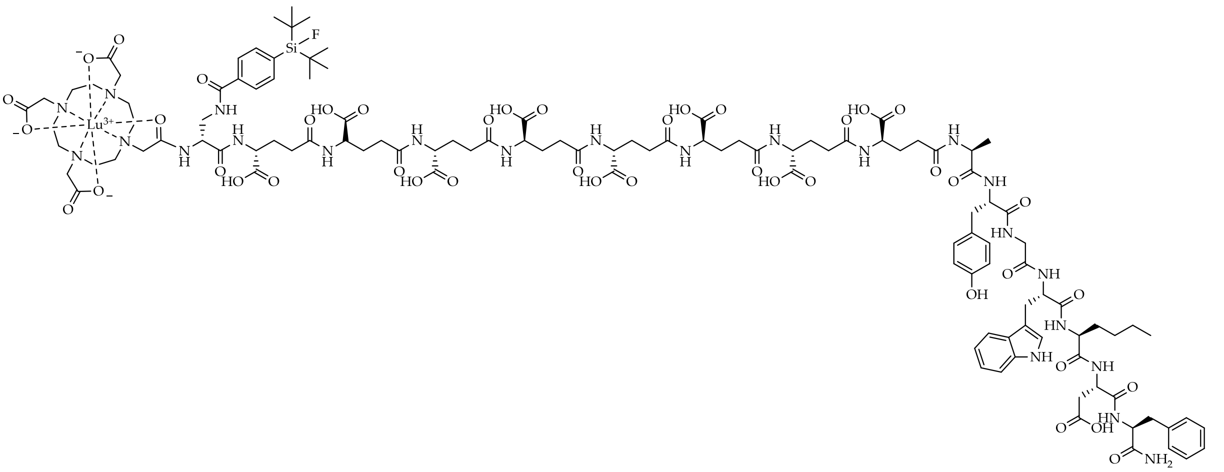

[natLu]Lu-DOTA-rhCCK-18: RP-HPLC (10→90% MeCN in H_2O with 0.1% TFA, 15 min, λ = 220 nm): t_R = 9.92 min, K' = 4.88; MS (ESI, positive): m/z calculated for $C_{116}H_{161}FLuN_{23}O_{43}Si$: 2811.8, found: m/z = 1405.8 $[M+2H]^{2+}$, 937.6 $[M+3H]^{3+}$.

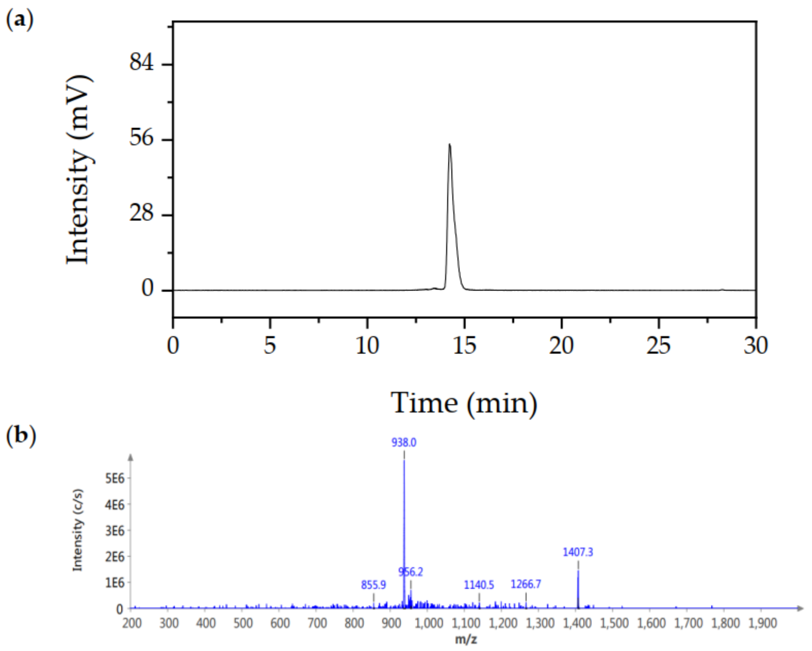

Figure S8. (**a**) Confirmation of peptide identity and integrity for [^{177}Lu]Lu-DOTA-rhCCK-18.2, as analyzed by analytical RP-HPLC (10→30% MeCN in H_2O + 0.1% TFA in 5 min, 30→60% MeCN in H_2O + 0.1% TFA in 15 min). (**b**) Mass spectrum of [^{nat}Lu]Lu-DOTA-rhCCK-18.2.

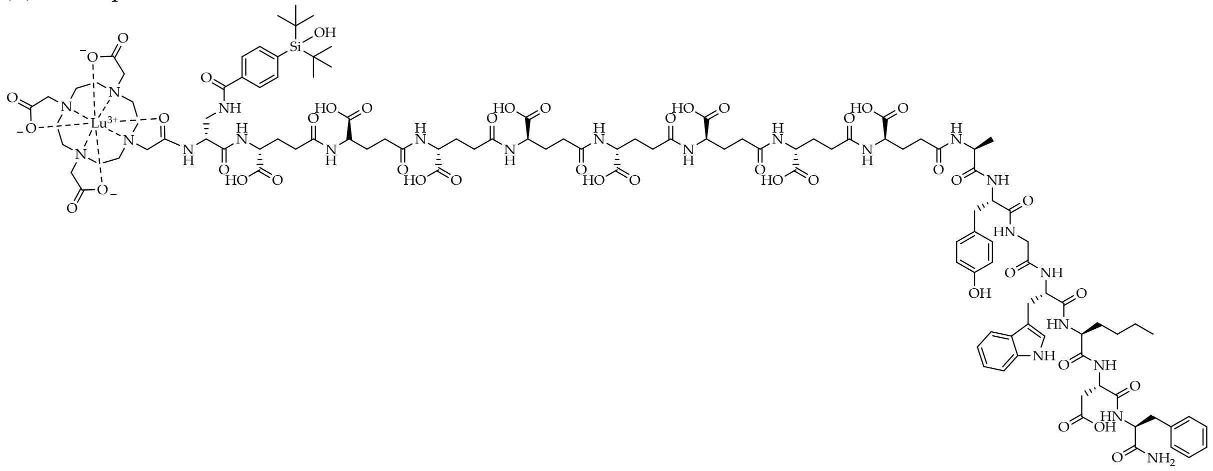

[$^{nat/177}$Lu]Lu-DOTA-rhCCK-18.2: RP-HPLC (10→30% MeCN in H_2O with 0.1% TFA, 5 min, 30→60% MeCN in H_2O with 0.1% TFA, 15 min, λ = 220 nm): t_R = 14.3 min, K' = 6.15; MS (ESI, positive): m/z calculated for $C_{116}H_{162}LuN_{23}O_{44}Si$: 2809.7, found: m/z = 1407.3 $[M+2H]^{2+}$, 938.0 $[M+3H]^{3+}$.

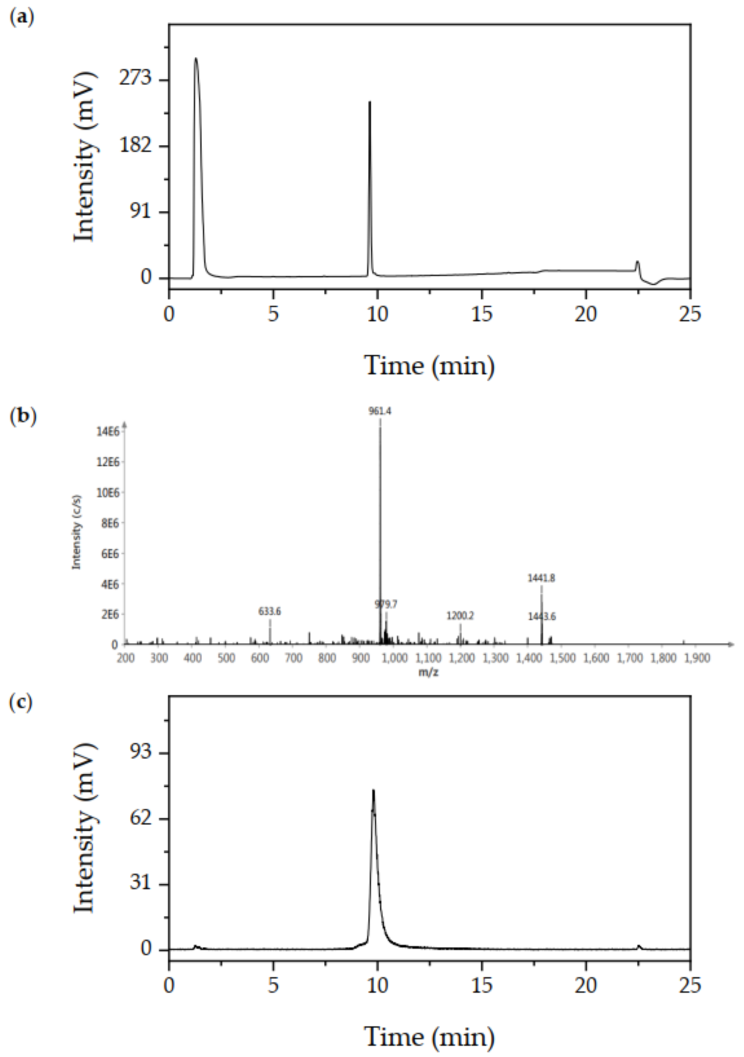

Figure S9. (**a**) Confirmation of peptide identity and integrity for [natLu]Lu-(*R*)-DOTAGA-rhCCK-18, as analyzed by analytical RP-HPLC (10→90% MeCN in H_2O + 0.1% TFA in 15 min). (**b**) Mass spectrum of [natLu]Lu-(*R*)-DOTAGA-rhCCK-18. (**c**) Confirmation of peptide identity and integrity for [177Lu]Lu-(*R*)-DOTAGA-rhCCK-18, as analyzed by analytical (radio-)RP-HPLC (10→90% MeCN in H_2O + 0.1% TFA in 15 min).

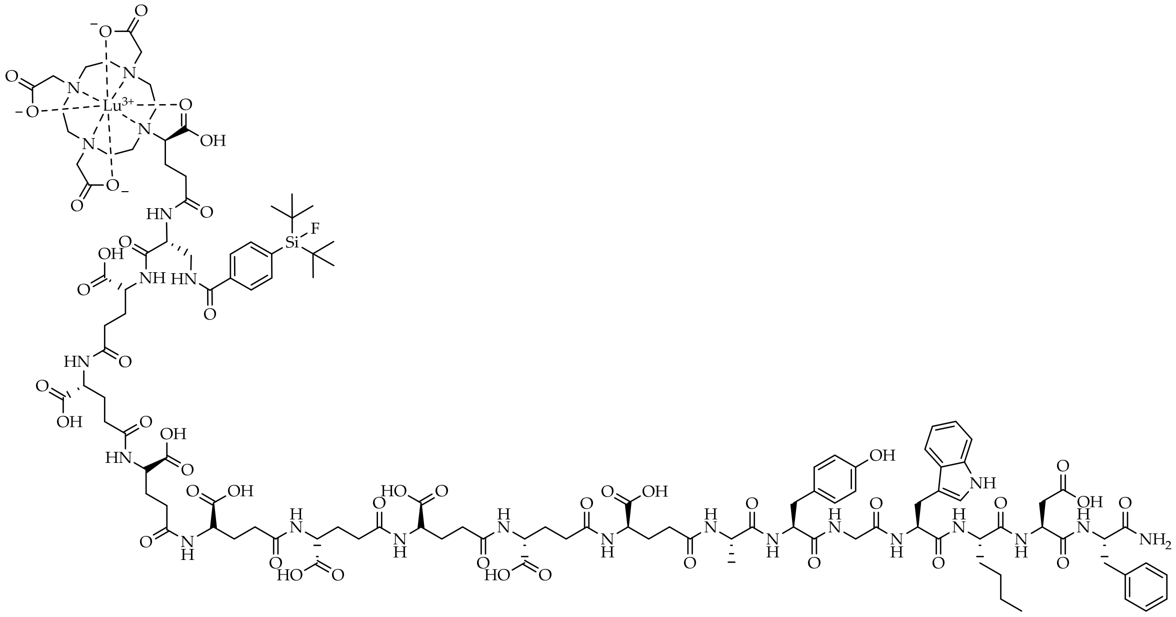

[^{nat}Lu]Lu-(R)-DOTAGA-rhCCK-18: RP-HPLC (10 → 90% MeCN in H_2O with 0.1% TFA, 15 min, λ = 220 nm): t_R = 9.50 min, K' = 4.63; MS (ESI, positive): m/z calculated for $C_{121}H_{165}FLuN_{23}O_{45}Si$: 2883.8, found: m/z = 1441.8 $[M+2H]^{2+}$, 961.4 $[M+3H]^{3+}$.

Analytical data of natGa-labeled minigastrin analogs

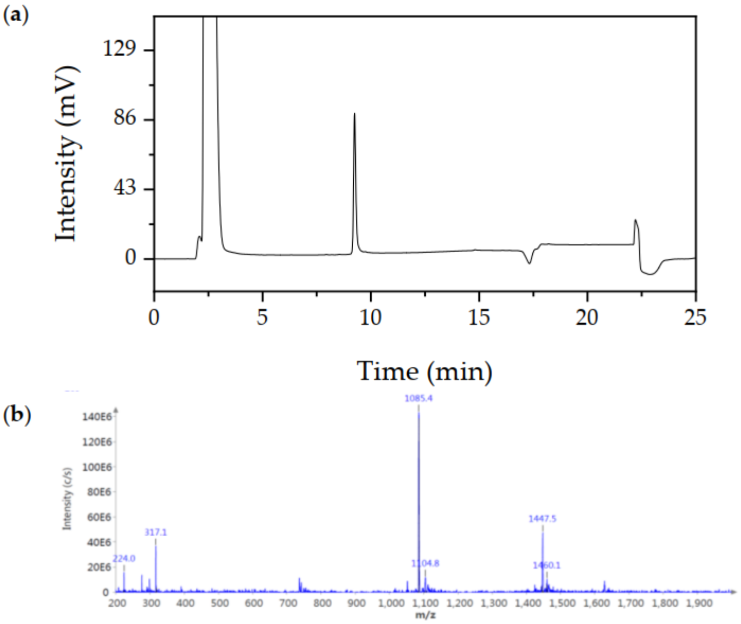

Figure S10. (**a**) Confirmation of peptide identity and integrity for [natGa]Ga-(*R*)-DOTAGA-PP-F11N, as analyzed by analytical RP-HPLC (10→90% MeCN in H_2O + 0.1% TFA in 15 min). (**b**) Mass spectrum of [natGa]Ga-(*R*)-DOTAGA-PP-F11N.

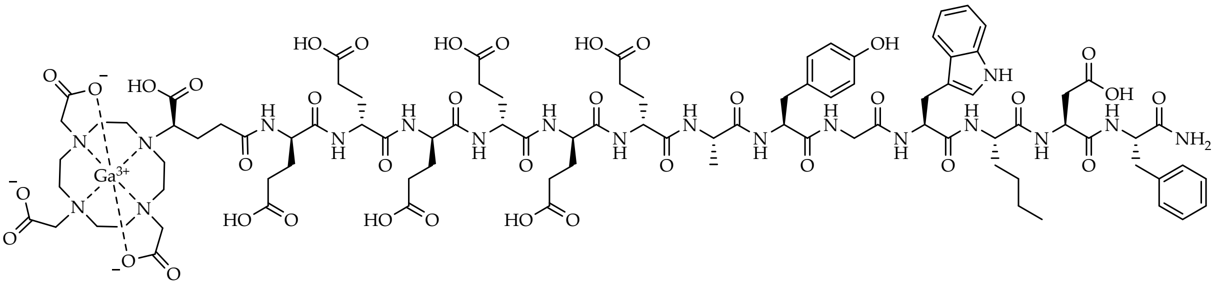

[natGa]Ga-(R)-DOTAGA-PP-F11N: RP-HPLC (10→70% MeCN in H_2O with 0.1% TFA, 15 min, λ = 220 nm): t_R = 9.3 min, K′ = 5.51; MS (ESI, positive): m/z calculated for $C_{93}H_{124}GaN_{19}O_{37}$: 2169.8, found: m/z = 1085.4 $[M+2H]^{2+}$.

(a)

(b)

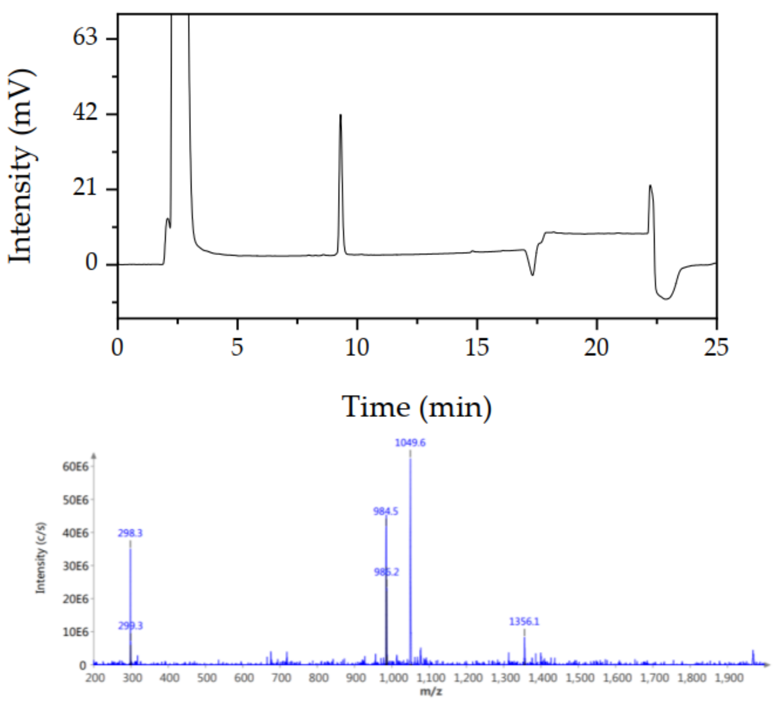

Figure S11. (**a**) Confirmation of peptide identity and integrity for [natGa]Ga-DOTA-PP-F11N, as analyzed by analytical RP-HPLC (10→90% MeCN in H_2O + 0.1% TFA in 15 min). (**b**) Mass spectrum of [natGa]Ga-DOTA-PP-F11N.

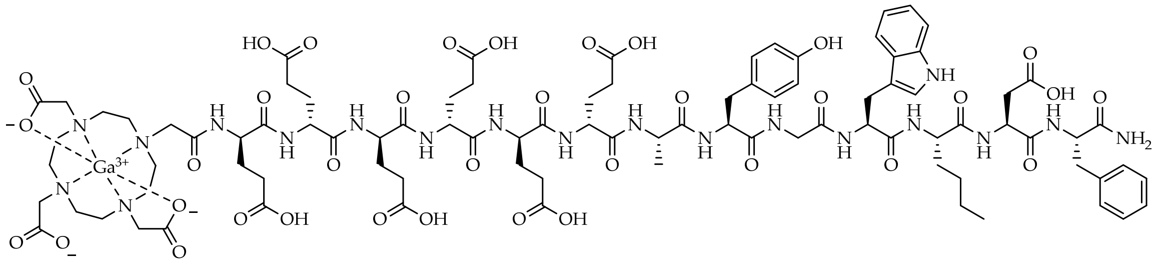

[natGa]Ga-DOTA-PP-F11N: RP-HPLC (10→70% MeCN in H_2O with 0.1% TFA, 15 min, λ = 220 nm): t_R = 9.3 min, K′ = 5.51; MS (ESI, positive): m/z calculated for $C_{90}H_{120}GaN_{19}O_{35}$: 2097.8, found: m/z = 1049.6 $[M+2H]^{2+}$.

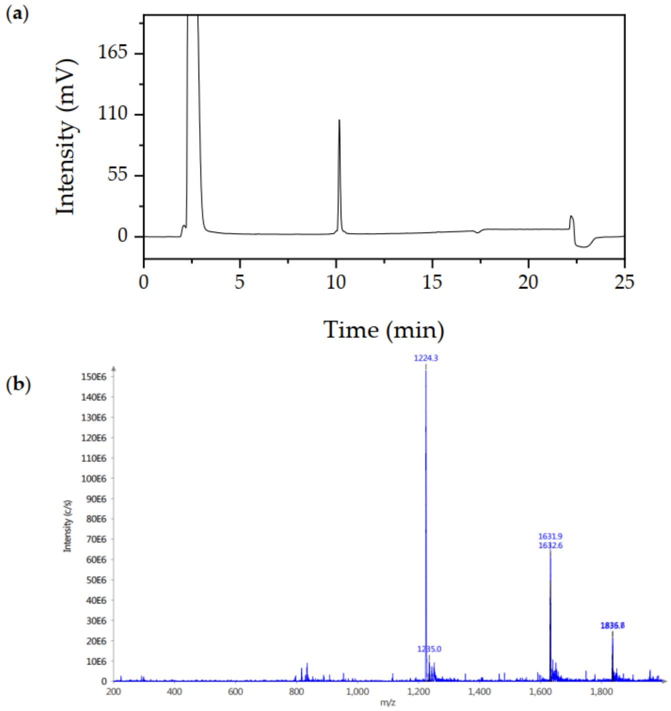

Figure S12. (**a**) Confirmation of peptide identity and integrity for [natGa]Ga-DOTA-rhCCK-16, as analyzed by analytical RP-HPLC (10→90% MeCN in H_2O + 0.1% TFA in 15 min). (**b**) Mass spectrum of [natGa]Ga-DOTA-rhCCK-16.

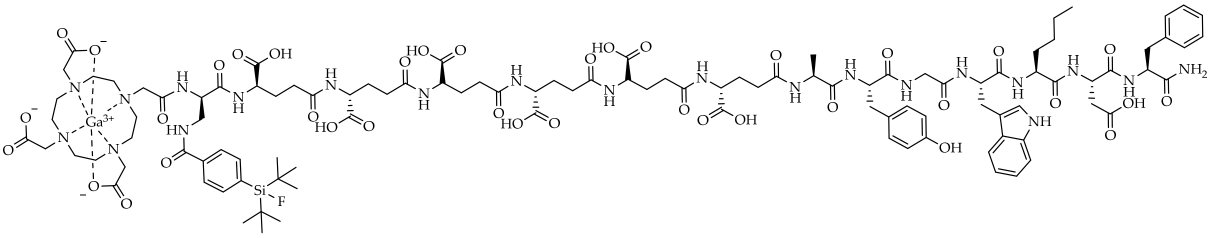

[natGa]Ga-DOTA-rhCCK-16: RP-HPLC (10→90% MeCN in H_2O with 0.1% TFA, 15 min, λ = 220 nm): t_R = 10.2 min, K′ = 5.05; MS (ESI, positive): m/z calculated for $c_{108}H_{147}FGaN_{21}O_{37}Si$: 2448.3, found: m/z = 1224.3 $[M+2H]^{2+}$.

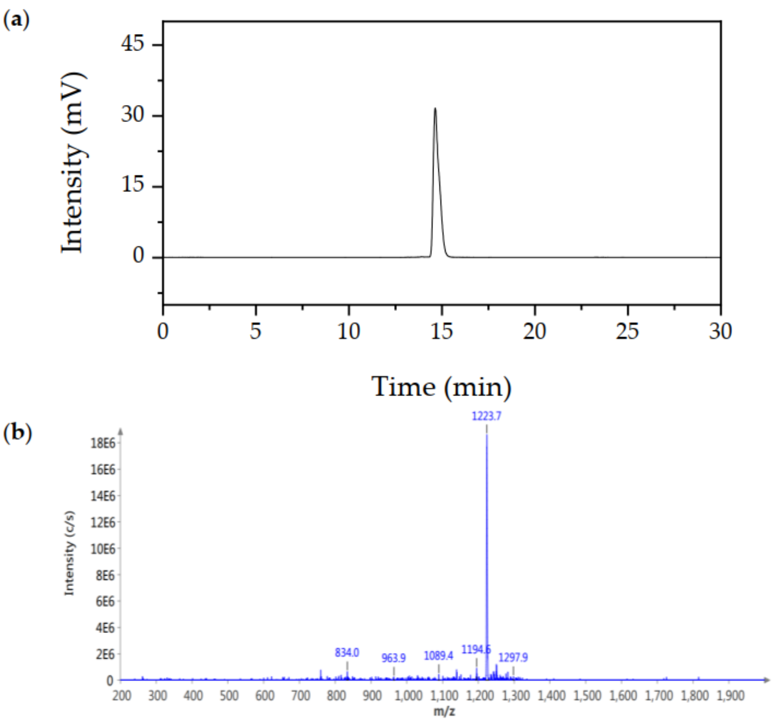

Figure S13. (**a**) Confirmation of peptide identity and integrity for [67Ga]Ga-DOTA-rhCCK-16.2, as analyzed by analytical RP-HPLC (10→30% MeCN in H_2O + 0.1% TFA in 5 min, 30→60% MeCN in H_2O + 0.1% TFA in 15 min). (**b**) Mass spectrum of [natGa]Ga-DOTA-rhCCK-16.2.

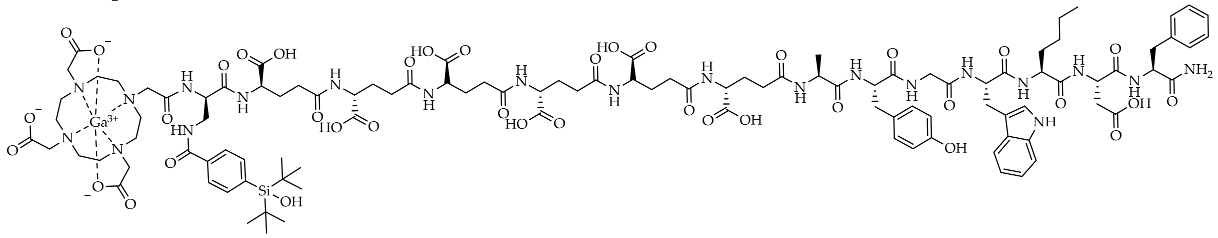

[nat/67Ga]Ga-DOTA-rhCCK-16.2: RP-HPLC (10→30% MeCN in H_2O with 0.1% TFA, 5 min, 30→60% MeCN in H_2O with 0.1% TFA, 15 min λ = 220 nm): t_R = 14.6 min, K' = 6.30; MS (ESI, positive): m/z calculated for $C_{108}H_{148}GaN_{21}O_{38}Si$: 2446.3, found: m/z = 1223.7 $[M+2H]^{2+}$.

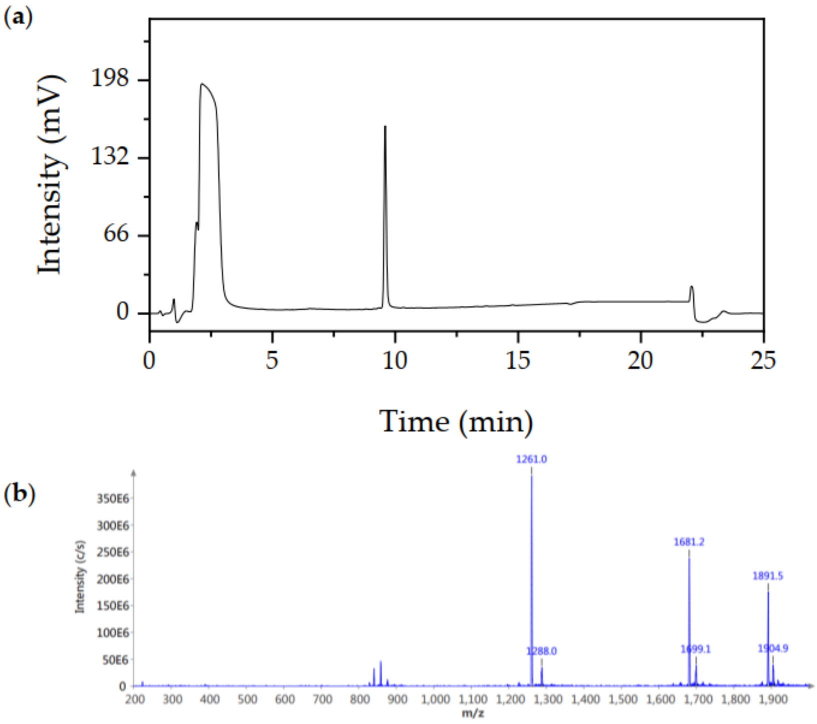

Figure S14. (**a**) Confirmation of peptide identity and integrity for [natGa]Ga-(*R*)-DOTAGA-rhCCK-16, as analyzed by analytical RP-HPLC (10→90% MeCN in H_2O + 0.1% TFA in 15 min). (**b**) Mass spectrum of [natGa]Ga-(*R*)-DOTAGA-rhCCK-16.

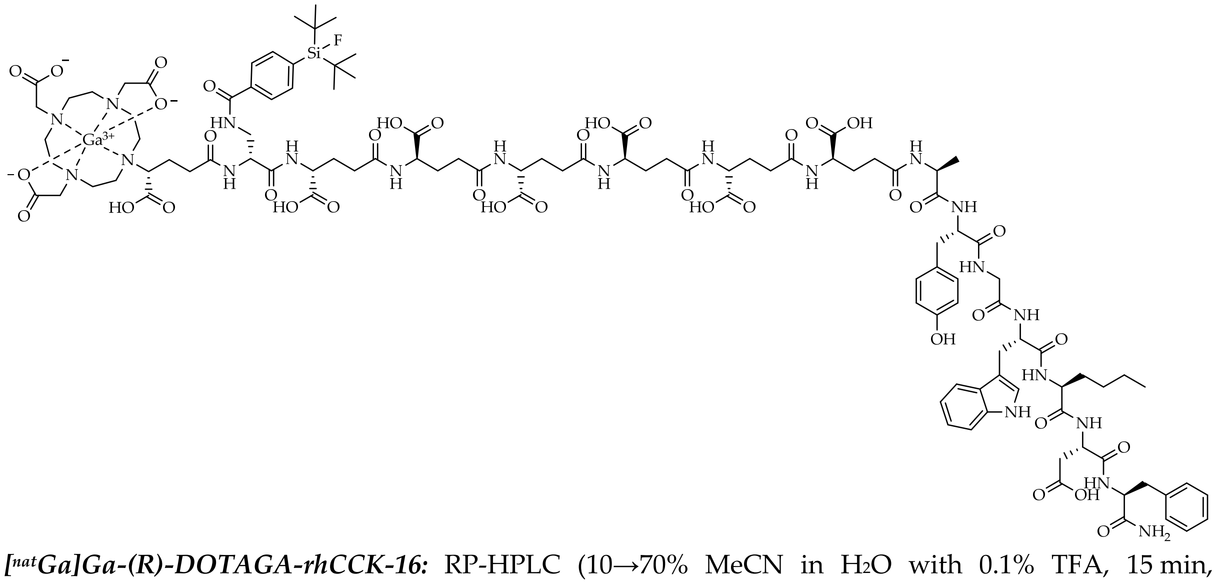

[natGa]Ga-(R)-DOTAGA-rhCCK-16: RP-HPLC (10→70% MeCN in H_2O with 0.1% TFA, 15 min, λ = 220 nm): t_R = 9.73 min, K′ = 5.08; MS (ESI, positive): m/z calculated for $C_{111}H_{151}FGaN_{21}O_{39}Si$: 2520.3, found: m/z = 1261.0 $[M+2H]^{2+}$.

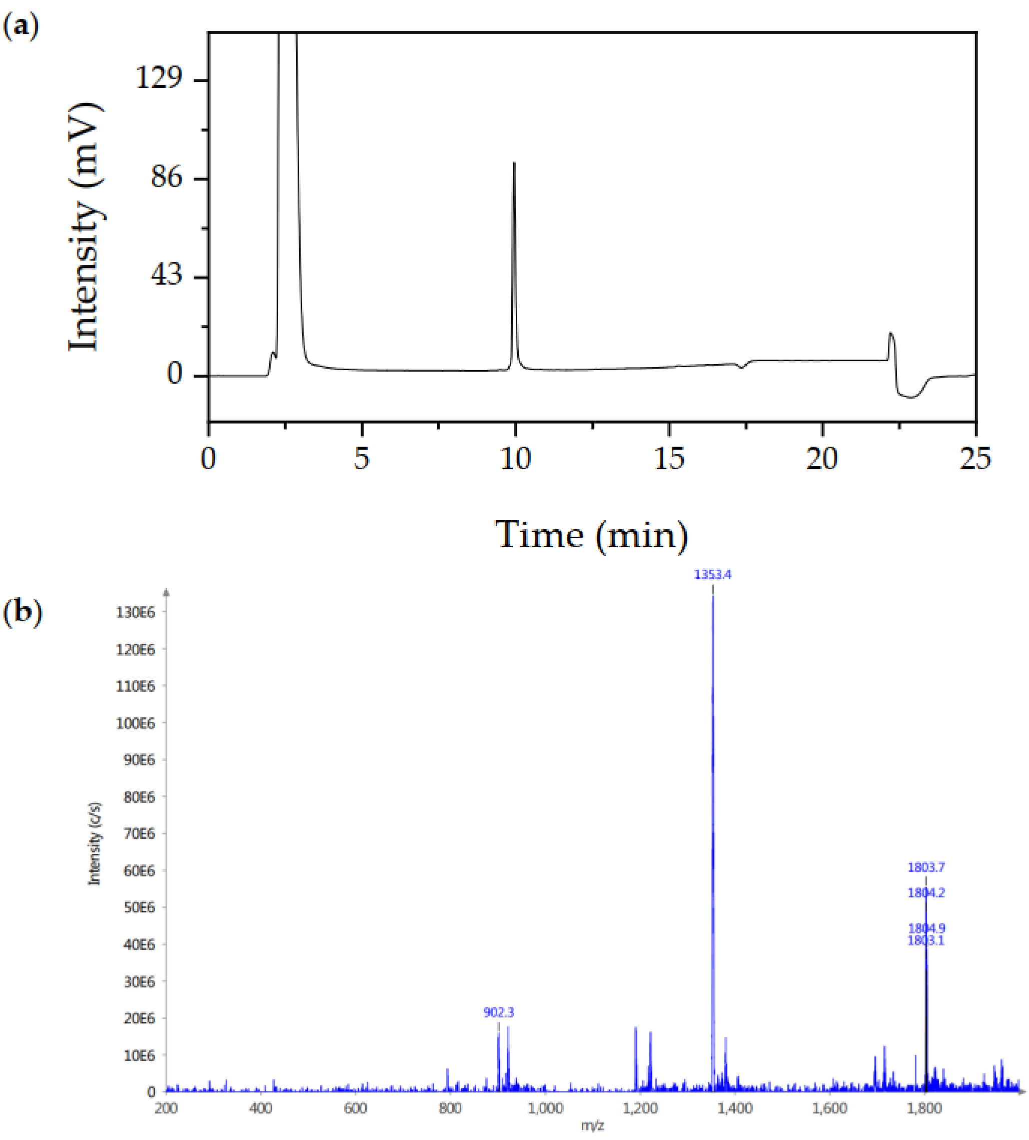

Figure S15. (**a**) Confirmation of peptide identity and integrity for [natGa]Ga-DOTA-rhCCK-18, as analyzed by analytical RP-HPLC (10→90% MeCN in H_2O + 0.1% TFA in 15 min). (**b**) Mass spectrum of [natGa]Ga-DOTA-rhCCK-18.

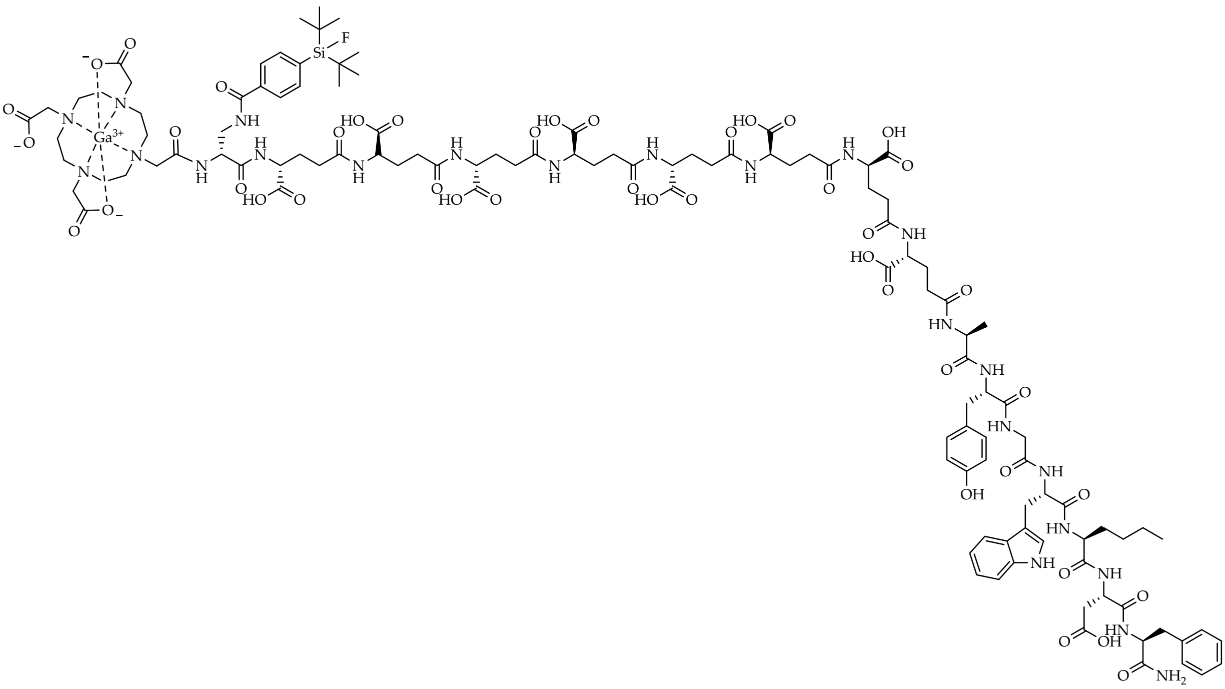

[natGa]Ga-DOTA-rhCCK-18: RP-HPLC (10→90% MeCN in H_2O with 0.1% TFA, 15 min, λ = 220 nm): t_R = 9.90 min, K' = 4.87; MS (ESI, positive): m/z calculated for $C_{118}H_{161}FGaN_{23}O_{43}Si$: 2706.5, found: m/z = 1353.4 $[M+2H]^{2+}$.

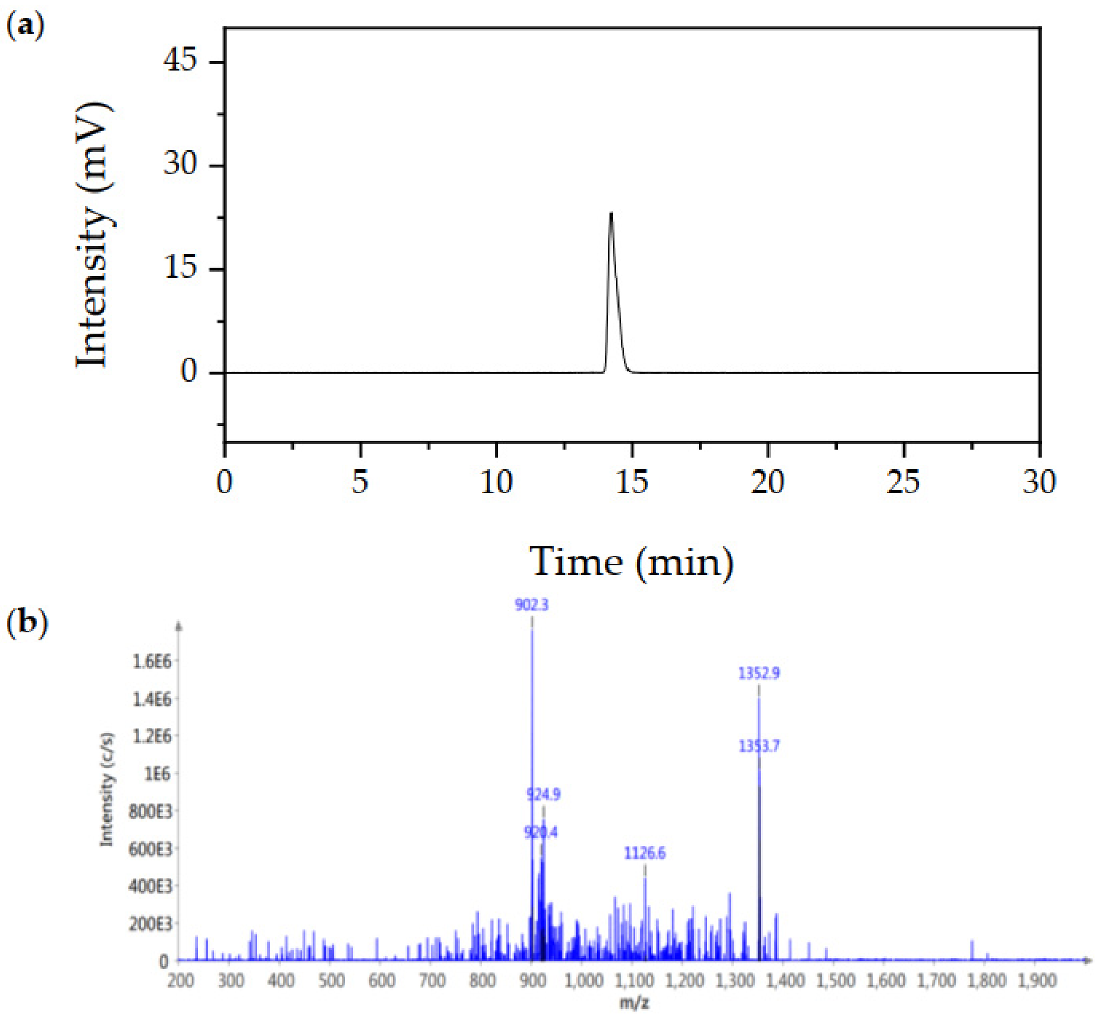

Figure S16. (**a**) Confirmation of peptide identity and integrity for [^{67}Ga]Ga-DOTA-rhCCK-18.2, as analyzed by analytical RP-HPLC (10→30% MeCN in H_2O + 0.1% TFA in 5 min, 30→60% MeCN in H_2O + 0.1% TFA in 5 min). (**b**) Mass spectrum of [^{nat}Ga]Ga-DOTA-rhCCK-18.2.

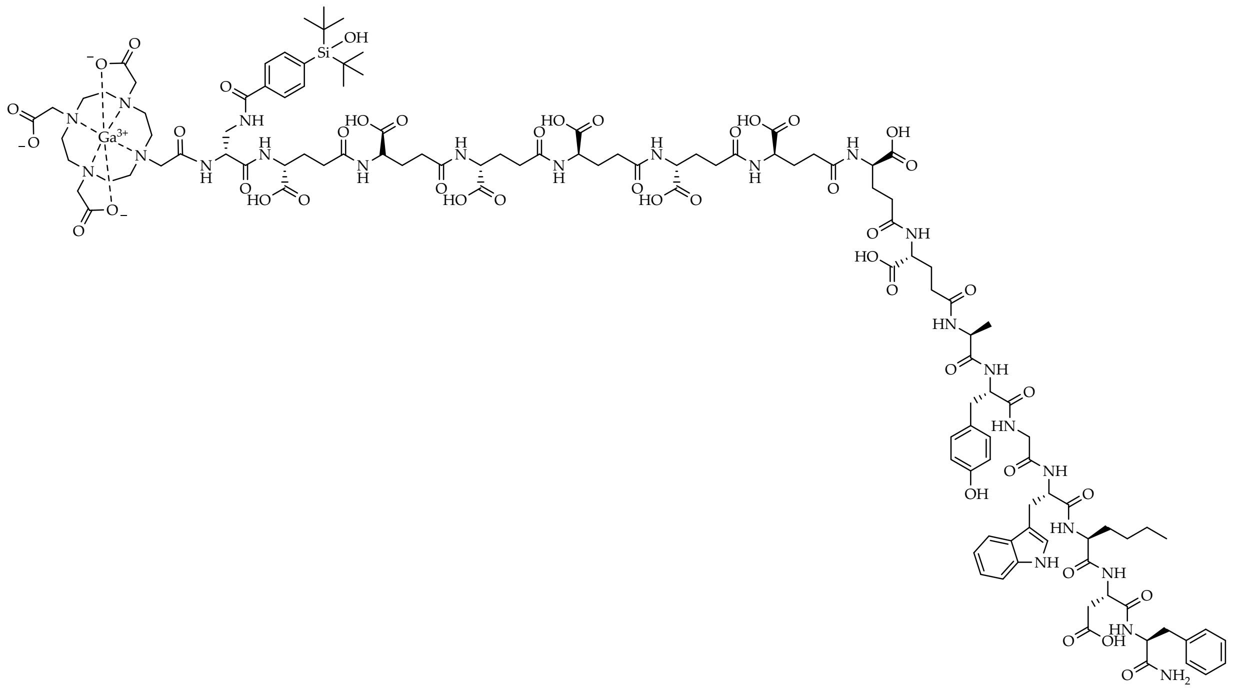

[nat/67Ga]Ga-DOTA-rhCCK-18.2: RP-HPLC (10→30% MeCN in H_2O with 0.1% TFA, 5 min, 30→60% MeCN in H_2O with 0.1% TFA, 15 min λ = 220 nm): t_R = 14.2 min, K' = 6.10; MS (ESI, positive): m/z calculated for $C_{118}H_{162}GaN_{23}O_{44}Si$: 2704.5, found: m/z = 1352.9 $[M+2H]^{2+}$, 902.3 $[M+3H]^{3+}$.

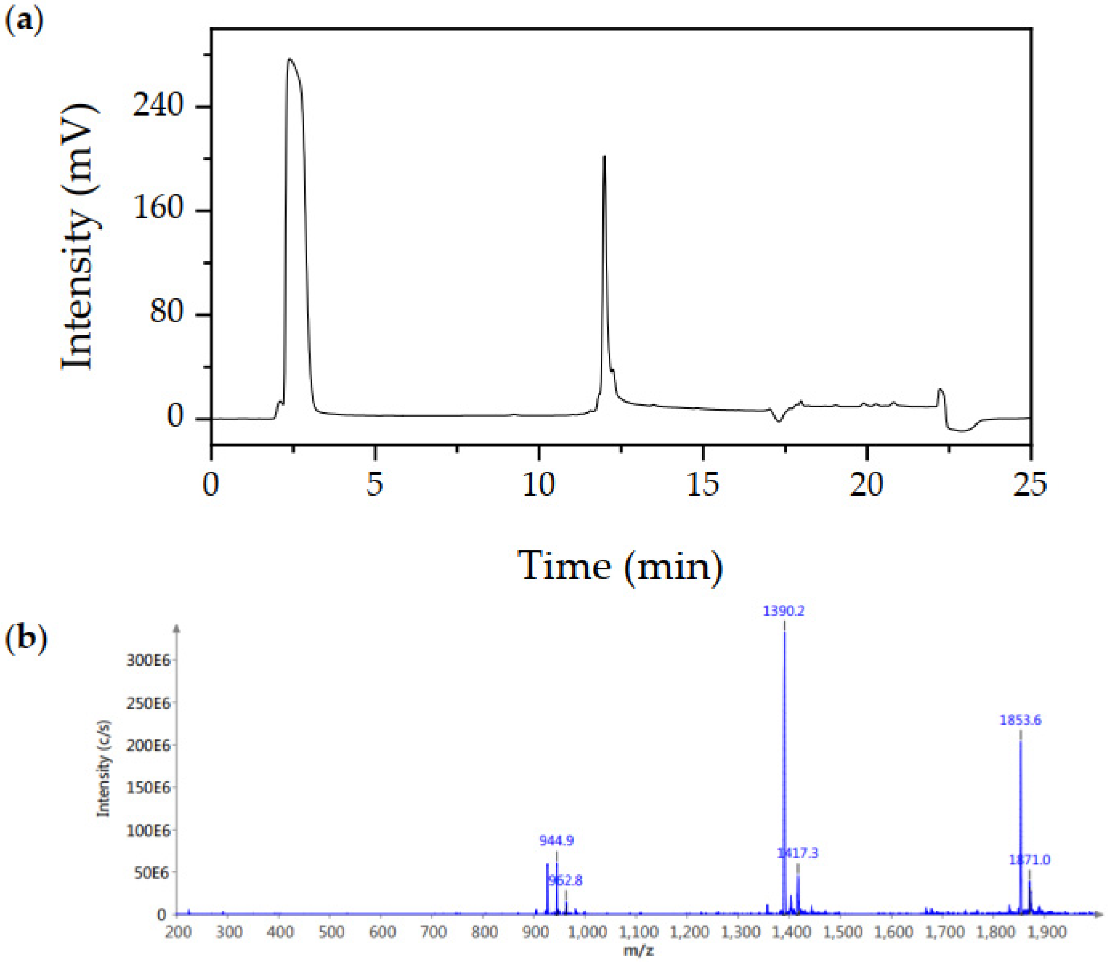

Figure S17. (**a**) Confirmation of peptide identity and integrity for [natGa]Ga-(*R*)-DOTAGA-rhCCK-18, as analyzed by analytical RP-HPLC (10→90% MeCN in H_2O + 0.1% TFA in 15 min). (**b**) Mass spectrum of [natGa]Ga-(*R*)-DOTAGA-rhCCK-18.

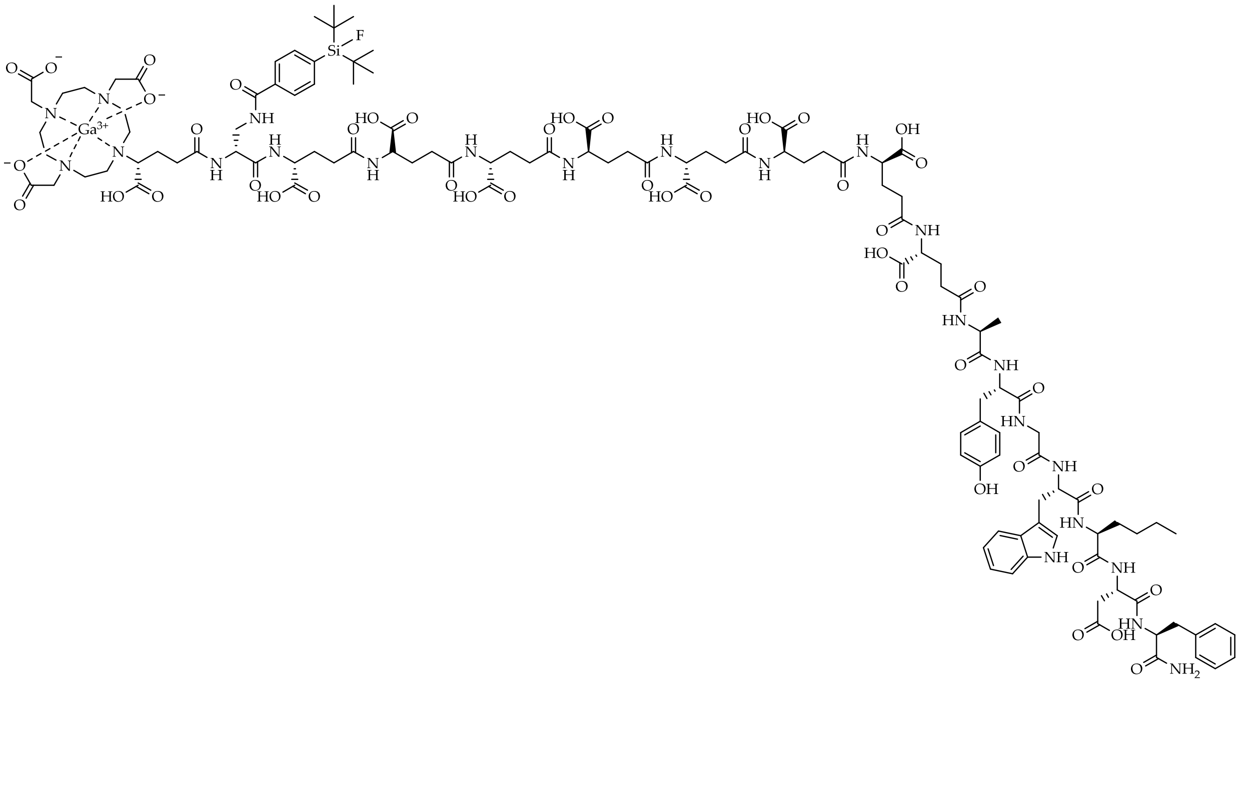

[^{nat}Ga]Ga-(R)-DOTAGA-rhCCK-18: RP-HPLC (10→70% MeCN in H_2O with 0.1% TFA, 15 min, λ = 220 nm): t_R = 12.0 min, K' = 6.11; MS (ESI, positive): m/z calculated for $C_{121}H_{165}FGaN_{25}O_{45}Si$: 2520.3, found: m/z = 1390.2 $[M+2H]^{2+}$.

SiFA hydrolysis

In order to hydrolyze the SiFA building block of the peptide precursors DOTA-rhCCK-16 and -18 to generate their SiOH-comprising analogs, DOTA-rhCCK-16.2 and 18.2, 1 eq. of each compound was treated with NaOH (15 eq., 12.5 mM), and the resulting solution was left at room temperature overnight. To terminate the reaction, HCl (15 eq., 25 mM) was added. Afterwards, the product was purified via RP-HPLC.

Labeling Procedures

^{18}F-Labeling

[^{18}F]fluoride (approx. 2-3 GBq) dissolved in H_2O was loaded onto a SEP-Pak® Light (46 mg) AccellTM Plus QMA cartridge (Waters GmbH, Eschborn, Germany) preconditioned with H_2O (10 mL). Afterwards, [^{18}F]fluoride was dried using 8 mL of anhydrous DMSO and inversely eluted from the cartridge with ammonium formate (250 µL, 50 mg NH_4HCOO dissolved in 500 µL anhydrous DMSO). Peptide precursor (1 nmol) was added to 50 µL eluate and the reaction mixture was heated to 60°C for 5 min. Afterwards, the reaction mixture was dissolved in PBS (10 mL, pH = 3) and the ^{18}F-labeled peptide was loaded onto an Oasis® HLB (30 mg) Light Cartridge (Waters GmbH, Eschborn, Germany) preconditioned with 10 mL EtOH and 10 mL H_2O. After washing the crude product with PBS (10 mL, pH = 7.4), the peptide was inversely eluted from the cartridge with 200 µL EtOH/H_2O (7/3). Radiochemical purity was determined using radio RP-HPLC and radio TLC (MeCN/PBS (pH = 7.4), 6/4 (*v*/*v*), +10% sodium acetate (2 M) + 1% TFA).

^{177}Lu-Labeling

For ^{177}Lu-labeling experiments, [^{177}Lu]$LuCl_3$ dissolved in hydrochloric acid (0.04 M, 40 GBq/mL) was acquired from ITM Isotope Technologies Munich SE (Garching, Germany). Radiolabeling of the peptide precursor (1 nmol) was performed at 90°C for 15 min in a NaOAc-buffered (1 M, pH = 5.5) hydrochloric acid (0.04 M) solution. After radiolabeling, a radiolysis quencher (sodium ascorbate, 1 M in H_2O) was added and radiochemical purity was determined via radio-RP-HPLC and radio-TLC (instant thin layer chromatography paper impregnated with silica gel (iTLC-SG, Agilent Technologies Inc., Folsom, United States); sodium citrate*1.5 H_2O (0.1 M)).

^{nat}Lu-Labeling

Quantitative ^{nat}Lu-labeling was conducted by stirring a solution of [^{nat}Lu]$LuCl_3$ (2.5 eq., 20 mM), peptide precursor (1 eq., 1 mM in DMSO) and Tracepur® H_2O at 90°C for 15 min. Confirmation of peptide integrity was performed via RP-HPLC and ESI-MS.

^{67}Ga-Labeling

^{67}Ga-Labeling experiments were conducted using 10-40 MBq of [^{67}Ga]$GaCl_3$ (Curium™ (Berlin, Germany), 4-(2-hydroxyethyl)-1-piperazineethanesulfonic acid-buffer (7 µL, 2.5 M, HEPES) and peptide precursor (1 nmol). The reaction mixture was stirred at 80 °C for 20 min. After radiolabeling, sodium ascorbate (1 M in H_2O) was added to prevent radiolysis, and radiochemical purity was determined via radio RP-HPLC and radio TLC (sodium citrate*1.5 H_2O (0.1 M)).

^{nat}Ga-Labeling

^{nat}Ga-Complexation of the CCK-2R ligands was accomplished by stirring a solution of [^{nat}Ga]$Ga(NO_3)_3$ (2.5 eq., 10 mM), peptide precursor (1 eq., 1 mM) and Tracepur® H_2O at 70 °C for 30 min. Confirmation of peptide integrity was performed via RP-HPLC and ESI-MS.

In Vitro Experiments

Cell Culture. CCK-2R-expressing rat pancreatic cancer cells AR42J (CLS GmbH, Eppelheim, Germany) were cultivated in monolayers in CELLSTAR® cell culture flasks acquired from Greiner Bio-One GmbH (Frickenhausen, Germany) at 37°C in a humidified atmosphere (5% CO_2) using a HERAcell 150i-Incubator (Thermo Fisher Scientific Inc., Waltham, United States). The nutrient medium RPMI 1640 medium, supplemented with 5 mM L-Gln 5 mL non-essential amino acids (100×) and 10% FCS, was used. Furthermore, a Dulbecco's PBS solution with 0.1% EDTA (*v/v*) was applied to detach the cells for cell passaging. The detached cells were counted using a Neubauer hemocytometer (Paul Marienfeld, Lauda-Königshofen, Germany). In addition, all operations under sterile conditions were accomplished using a MSC-Advantage safety workbench (Thermo Fisher Scientific Inc., Waltham, United States).

Determination of IC_{50}. AR42J cells (2.0 × 10^5 cells/well) were seeded into 24-well plates 24 ± 2 h prior to testing using 1 mL of nutrient medium (RPMI 1640, 5 mM L-Gln, 5 mL non-essential amino acids (100×), 10% FCS). Cells were incubated at 37°C in a humidified atmosphere (5% CO_2).
After the removal of the medium, each well was washed with 500 µL PBS. For the cell-based assay, 200 µL of the nutrient medium (+5% BSA), [^{177}Lu]Lu-DOTA-PP-F11N (25 µL, 0.3 pmol) as a radiolabeled reference and 25 µL of the peptide of interest in increasing concentrations (10^{-10} to 10^{-4} M) were added to the cells in triplicate. Thereafter, the assay was incubated for 3 h at 37°C and the supernatant was collected. The cells were washed with 300 µL PBS and the collected supernatant fractions were unified. After lysis of the cells with NaOH (300 µL, 1 N) for 15 min, the respective wells were washed with NaOH (300 µL, 1 N) and both fractions were unified. The radioactivity of both the supernatant and the lysed fractions were quantified using a γ-counter (PerkinElmer Inc., Waltham, United States). The obtained data were evaluated via the GraphPad PRISM software (GraphPad Software Inc., La Jolla, United States), which calculates the half-maximal inhibitory concentration (IC_{50}) of the peptides.

Internalization Studies. For the determination of the internalization kinetics of the various peptides, AR42J cells (3.0 × 10^5 cells/well) were seeded into polylysine-coated 24-well plates, adding 1 mL of nutrient medium. Afterwards, the cells were incubated for 24 ± 2 h at 37°C in a humidified atmosphere (5% CO_2).

On the day of the experiment, the medium was removed, and each well was washed with nutrient medium (300 µL). Afterwards 200 µL of nutrient medium, 25 µL of the ^{177}Lu-labeled peptide (0.3 pmol, $n = 6$) and either 25 µL of nutrient medium for internalization studies ($n = 3$) or 25 µL of [^{nat}Lu]Lu-DOTA-PP-F11N (10 µmol) for competition studies ($n = 3$) were added. Thereafter, the assay was incubated for various time points (1, 2, 4 and 6 h) at 37°C in a humidified atmosphere (5% CO_2). After incubation, the cells were put on ice for at least 1 min to stop internalization kinetics and the supernatant was collected. Then, the cells were washed with an ice-cold nutrient medium (300 µL) and both fractions were unified. In order to displace the peptides from the cell membrane, 300 µL of an ice-cold glycine buffer (1 M, pH = 2.2) were added and the cells were incubated on ice for 15 min. Afterwards, the supernatant was collected, and the cells were washed with an ice-cold glycine buffer (300 µL, 1 M, pH = 2.2) while both fractions were unified. After lysis of the cells with NaOH (300 µL, 1 N) for 15 min, the respective wells were washed with NaOH (300 µL, 1 N) and both fractions were unified. The radioactivity of the supernatant, the acid wash and the lysed fractions were quantified using a γ-counter.

Lipophilicity Studies. Lipophilicity (depicted as octanol-phosphate-buffered saline solution (PBS, pH = 7.4) distribution coefficient, log$D_{7.4}$) was determined by dissolving the ^{177}Lu-labelled peptide (approx. 1 MBq) in a mixture (1/1, *v/v*) of n-octanol and PBS. The suspension was vortexed in a reaction vial (1.5 mL) for 3 min at room temperature and the vial was centrifuged at 9000 rpm for 5 min (Biofuge 15, Heraus Sepatech GmbH, Osterode, Germany). Amounts of 200 µL aliquots of both layers were measured separately in a γ-counter (Perkin Elmer, Waltham, MA, USA). The experiment was repeated at least five times.

Stability Studies in Human Serum. The ^{177}Lu- as well as ^{67}Ga-labeled CCK-2R ligands (1 nmol, approx. 5 MBq) were incubated for 24 h in human serum (200 µL) at 37 °C. After incubation, ice-cold EtOH (125 µL) and MeCN (375 µL) were added and the suspension was centrifuged for 5 min at 5000 rpm. Then, the supernatant was transferred into new vials and centrifuged for another 5 min at 5000 rpm. After separating the precipitate from the solution, the stability of the ligands was determined via RP-HPLC chromatography (10→30% MeCN in H_2O + 0.1% TFA in 5 min, 30→60% MeCN in H_2O + 0.1% TFA in 15 min).

Table S1. Affinity data ($n = 3$) of the compounds evaluated, determined on AR42J cells (2.0 x 10^5 cells/well) with [^{177}Lu]Lu-DOTA-PP-F11N (0.3 pmol/well) as radiolabeled reference (3 h, 37°C, RPMI 1640, 5 mM L-Gln, 5 mL non-essential amino acids (100x), 10% FCS + 5% BSA (*v*/*v*)).

**peptide**	***IC*₅₀** (nM)<br>^{nat}Lu-labeled	***IC*₅₀** (nM)<br>^{nat}Ga-labeled
DOTA-PP-F11N	12.8 ± 2.8	17.0 ± 0.8
(*R*)-DOTAGA-PP-F11N	16.8 ± 2.8	13.9 ± 0.7
(*R*)-DOTAGA-rhCCK-16	20.4 ± 2.7	62.3 ± 6.9
DOTA-rhCCK-16	7.54 ± 0.26	16.8 ± 0.9
(*R*)-DOTAGA-rhCCK-18	20.4 ± 2.0	52.9 ± 6.0
DOTA-rhCCK-18	4.71 ± 0.62	13.7 ± 1.3

Table S2. Receptor-mediated internalization values (37 °C, RPMI 1640, 5 mM L-Gln, 5 mL non-essential amino acids (100x), 10% FCS, 0.25 pmol/well) determined as percentages (%) of the applied activity of [^{177}Lu]Lu-(*R*)-DOTAGA-rhCCK-18 as well as [^{177}Lu]Lu-DOTA-rhCCK-16 and -18 using AR42J cells (3.0 x 10^5 cells/well) at different time points (1, 2, 4 and 6 h). Data are corrected for non-specific binding (10 µmol, [^{nat}Lu]Lu-DOTA-PP-F11N).

**Peptide**	**Internalization Values** (%)				**Internalization Values*** (%F11N)
	**1 h**	**2 h**	**4 h**	**6 h**	**6 h**
[^{177}Lu]Lu-DOTA-PP-F11N	6.44 ± 0.32	10.1 ± 0.4	17.5 ± 1.0	22.4 ± 0.6	-
[$^{nat/177}$Lu]Lu-DOTA-rhCCK-16	19.5 ± 2.3	33.1 ± 1.7	43.9 ± 0.7	51.1 ± 1.7	215 ± 7
[$^{nat/177}$Lu]Lu-DOTA-rhCCK-18	15.4 ± 1.4	24.1 ± 1.6	41.6 ± 2.6	54.8 ± 1.5	244 ± 7
[^{177}Lu]Lu-(*R*)-DOTAGA-rhCCK-16	7.63 ± 0.13	14.0 ± 1.3	23.2 ± 2.7	32.2 ± 2.1	135 ± 9
[$^{nat/177}$Lu]Lu-(*R*)-DOTAGA-rhCCK-18	8.75 ± 1.43	16.8 ± 1.7	29.3 ± 1.3	36.8 ± 2.0	154 ± 8

* Internalization values are listed relative to the reference DOTA-PP-F11N

Table S3. Amounts of intact peptides and their analogs containing a hydrolyzed SiFA (=SiOH) moiety (n = 3) of the compounds evaluated, determined in human serum after incubation at 37°C for 24 h.

**Peptide**	Intact peptide (%)	SiOH-containing analog (%)
[^{67}Ga]Ga-DOTA-rhCCK-16	39.6 ± 7.9	56.5 ± 8.0
[^{177}Lu]Lu-DOTA-rhCCK-16	21.8 ± 0.8	69.1 ± 1.0
[^{67}Ga]Ga-DOTA-rhCCK-18	43.7 ± 6.9	53.6 ± 6.6
[^{177}Lu]Lu-DOTA-rhCCK-18	25.3 ± 3.1	67.7 ± 3.1

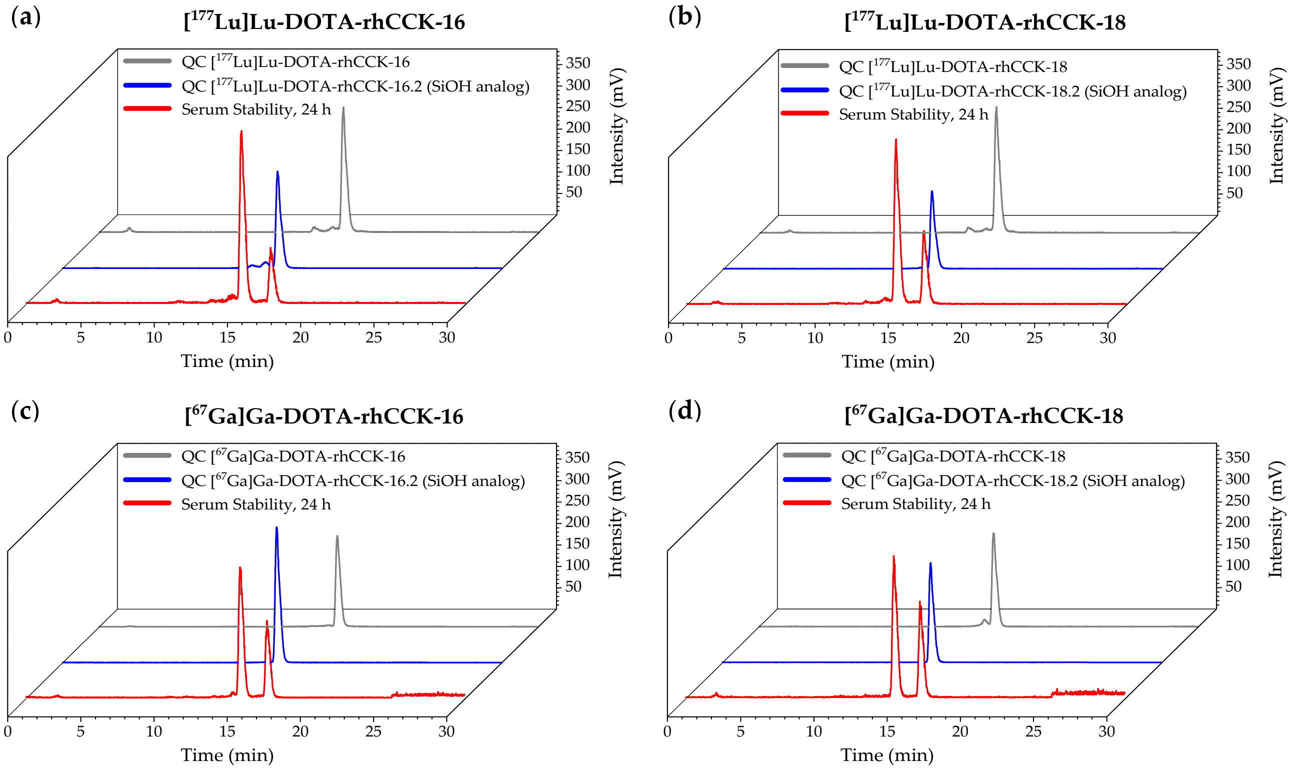

Figure S18. Stability studies of (a) [^{177}Lu]Lu-DOTA-rhCCK-16, (b) [^{177}Lu]Lu-DOTA-rhCCK-18, (c) [^{67}Ga]Ga-DOTA-rhCCK-16, and (d) [^{67}Ga]Ga-DOTA-rhCCK-18 in human serum (37°C, 24 h), as analyzed by analytical RP-HPLC (10→30% MeCN in H_2O + 0.1% TFA in 5 min, 30→60% MeCN in H_2O + 0.1% TFA in 5 min). The chromatograms of the respective compounds after incubation in human serum (37°C, 24 h) are depicted in red. Quality controls of the intact compounds comprising a SiFA moiety are depicted in gray and quality controls of the SiOH-comprising analogs ("hydrolyzed SiFA moiety") are depicted in blue.

Table S4. Biodistribution data of [^{177}Lu]Lu-DOTA-rhCCK-18 in selected organs at 1 and 24 h p.i. in AR42J tumor-bearing CB17-SCID mice (100 pmol each). Data are expressed as %ID/g, mean ± SD (*n* = 4). Biodistribution data of [^{177}Lu]Lu-DOTA-rhCCK-18 (100 pmol) co-injected with [^{177}Lu]Lu-DOTA-MGS5 in selected organs at 24 h p.i. in AR42J tumor-bearing CB17-SCID mice. Data are expressed as %ID/g, mean ± SD (*n* = 2).

Organ	[^{177}Lu]Lu-DOTA-rhCCK-18 (1 h p.i.)	[^{177}Lu]Lu-DOTA-rhCCK-18 (24 h p.i.)	[^{177}Lu]Lu-DOTA-rhCCK-18 (24 h p.i.) competition studies
Blood	2.56 ± 0.60	0.02 ± 0.01	0.02 ± 0.00
Heart	0.94 ± 0.02	0.08 ± 0.02	0.08 ± 0.01
Lung	2.05 ± 0.20	0.18 ± 0.15	0.76 ± 0.17
Liver	1.15 ± 0.18	0.53 ± 0.07	1.51 ± 0.02
Spleen	0.60 ± 0.08	0.22 ± 0.02	0.95 ± 0.14
Pancreas	1.22 ± 0.21	0.34 ± 0.09	0.22 ± 0.02
Stomach	4.25 ± 0.45	4.28 ± 1.05	0.18 ± 0.02
Intestine	0.61 ± 0.11	0.21 ± 0.02	0.17 ± 0.06
Kidney	97.2 ± 14.0	134 ± 18	193 ± 15
Adrenal	0.52 ± 0.07	0.37 ± 0.12	5.02 ± 4.64
Muscle	0.38 ± 0.03	0.08 ± 0.06	0.07 ± 0.04
Bone	0.48 ± 0.02	0.18 ± 0.03	0.22 ± 0.06
Tumor	24.1 ± 4.2	25.4 ± 4.7	1.75 ± 0.26

Table S5. Tumor-to-background ratios of [^{177}Lu]Lu-DOTA-rhCCK-18, [^{177}Lu]Lu-(*R*)-DOTAGA-rhCCK-16 and [^{177}Lu]Lu-(*R*)-DOTAGA-rhCCK-9 for the selected organs of AR42J tumor-bearing CB17-SCID mice at 24 h p.i. (100 pmol each). Data are expressed as mean ± SD (*n* = 4).

Organ	[^{177}Lu]Lu-DOTA-rhCCK-18	[^{177}Lu]Lu-(*R*)-DOTAGA-rhCCK-16	[^{177}Lu]Lu-(*R*)-DOTAGA-rhCCK-18
Blood	1630 ± 823	1067 ± 112	421 ± 152
Heart	312 ± 58	144 ± 35	72.4 ± 4.0
Lung	236 ± 118	176 ± 59	79.9 ± 29.9
Liver	48.1 ± 6.7	14.5 ± 5.1	7.24 ± 2.54
Spleen	115 ± 15	25.3 ± 5.9	19.8 ± 11.7
Pancreas	78.8 ± 17.5	46.2 ± 19.6	27.6 ± 14.0
Stomach	6.58 ± 2.77	4.59 ± 1.37	3.64 ± 0.99
Intestine	124 ± 30	81.9 ± 20.9	25.3 ± 8.3
Kidney	0.19 ± 0.01	0.18 ± 0.04	0.08 ± 0.01
Adrenal	73.3 ± 13.4	28.9 ± 18.5	26.8 ± 20.3
Muscle	650 ± 542	321 ± 77	307 ± 149
Bone	151 ± 48	96.8 ± 28.6	9.57 ± 4.67

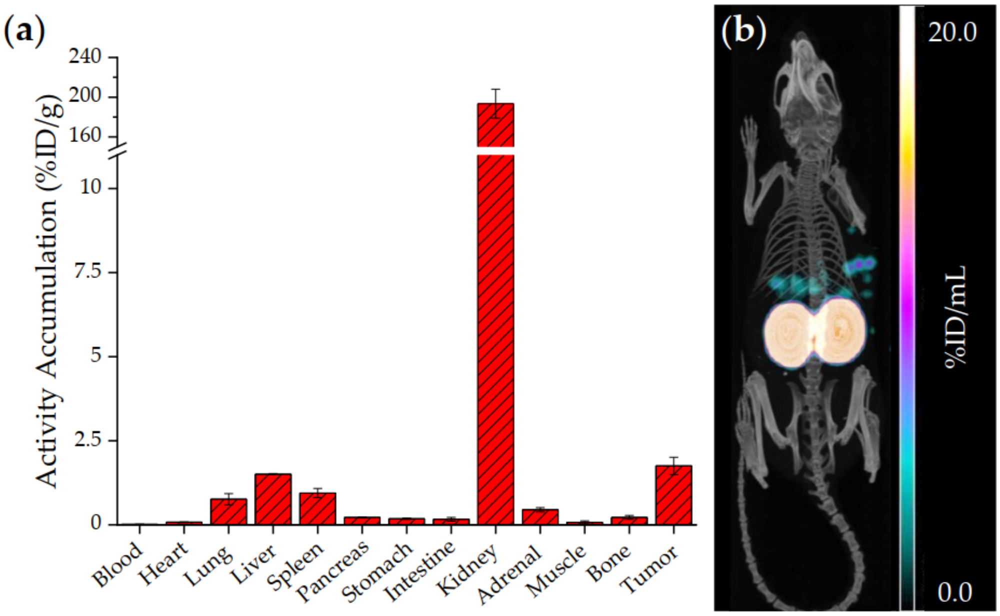

Figure S19. (**a**) Biodistribution and (**b**) a representative *μ*SPECT/CT image of [^{177}Lu]Lu-DOTA-rhCCK-18 (100 pmol) co-injected with [^{nat}Lu]Lu-DOTA-MGS5 (40 nmol) in selected organs (%ID/g) at 24 h p.i. in AR42J tumor-bearing CB17-SCID mice. Data is expressed as mean ± SD (*n* = 2).
